# Psychiatric comorbidity and disparities in the invasive management of acute myocardial infarction: a national analysis, 2016–23

**DOI:** 10.1093/ehjopen/oeag171

**Published:** 2026-10-13

**Authors:** Viswanadh Polana, Vikasa Godishala, Divyasri Koneru, Siddharth Karipineni, Simran Tanna, Nishant Patel, Dhairya Nanavaty, Sohrab Singh, Cesar Ayala-Rodriguez, Sarath Reddy

**Affiliations:** Internal Medicine Department, The Brooklyn Hospital Center, 121 DeKalb Avenue, Brooklyn, NY 11201, USA; Rutgers School of Health Professions, 65 Bergen Street, Newark, NJ 07107, USA; St Francis Medical Center, 3630 E. Imperial Highway, Lynwood, CA 90262, USA; Internal Medicine Department, The Brooklyn Hospital Center, 121 DeKalb Avenue, Brooklyn, NY 11201, USA; Internal Medicine Department, The Brooklyn Hospital Center, 121 DeKalb Avenue, Brooklyn, NY 11201, USA; Mount Sinai Hospital, 3201 Kings Highway, Brooklyn, NY 11234, USA; Mount Sinai Hospital, 3201 Kings Highway, Brooklyn, NY 11234, USA; Mount Sinai Hospital, 3201 Kings Highway, Brooklyn, NY 11234, USA; Internal Medicine Department, The Brooklyn Hospital Center, 121 DeKalb Avenue, Brooklyn, NY 11201, USA; Internal Medicine Department, The Brooklyn Hospital Center, 121 DeKalb Avenue, Brooklyn, NY 11201, USA

**Keywords:** Mental health disorders, Acute myocardial infarction, Schizophrenia, Coronary revascularization, Mechanical circulatory support, Health disparities

## Abstract

**Aims:**

Mental health disorders (MHDs) are common in acute myocardial infarction (AMI) and have been associated with less intensive cardiac care. We examined trends in five MHDs and their adjusted associations with in-hospital mortality, mechanical circulatory support (MCS), and coronary revascularization.

**Methods and results:**

We analysed the National Inpatient Sample, 2016–23, including adult hospitalizations with a principal diagnosis of AMI [ICD-10-CM I21.x/I22.x, excluding type 2 myocardial infarction (MI)]; five MHDs were identified from secondary diagnoses. Survey-weighted logistic regression produced adjusted odds ratios (aORs), adjusting for demographics, hospital characteristics, MI type, and 19 comorbidities, with all five MHDs entered simultaneously. Sensitivity analyses were restricted to hospitalizations without prior MI, percutaneous coronary intervention, or coronary artery bypass grafting. Among 970 969 AMI hospitalizations, prevalence rose for every MHD except schizophrenia/psychotic disorders, most steeply for PTSD (Post-traumatic stress disorder). Schizophrenia/psychotic disorders had the lowest adjusted odds of revascularization [aOR, 0.56; 95% confidence interval, 0.53–0.60] and invasive management (0.52; 0.49–0.55), followed by bipolar disorder (revascularization 0.81; 0.78–0.85); MCS associations were attenuated after adjustment. Restricted to those without prior coronary disease, the schizophrenia deficit was larger (0.54; 0.50–0.57) than among those with prior coronary disease (0.67; 0.60–0.74; interaction *P* < 0.001). Depression, anxiety, bipolar disorder, and PTSD were associated with lower adjusted in-hospital mortality (all *P* < 0.001); schizophrenia/psychotic disorders showed no significant difference (1.11; 0.98–1.26; *P* = 0.09).

**Conclusion:**

Schizophrenia and other psychotic disorders were consistently associated with markedly lower odds of invasive evaluation and coronary revascularization after AMI, with the largest deficit at first presentation. These findings highlight a potentially modifiable gap in cardiovascular care.

## Graphical Abstract

**Graphical Abstract:**
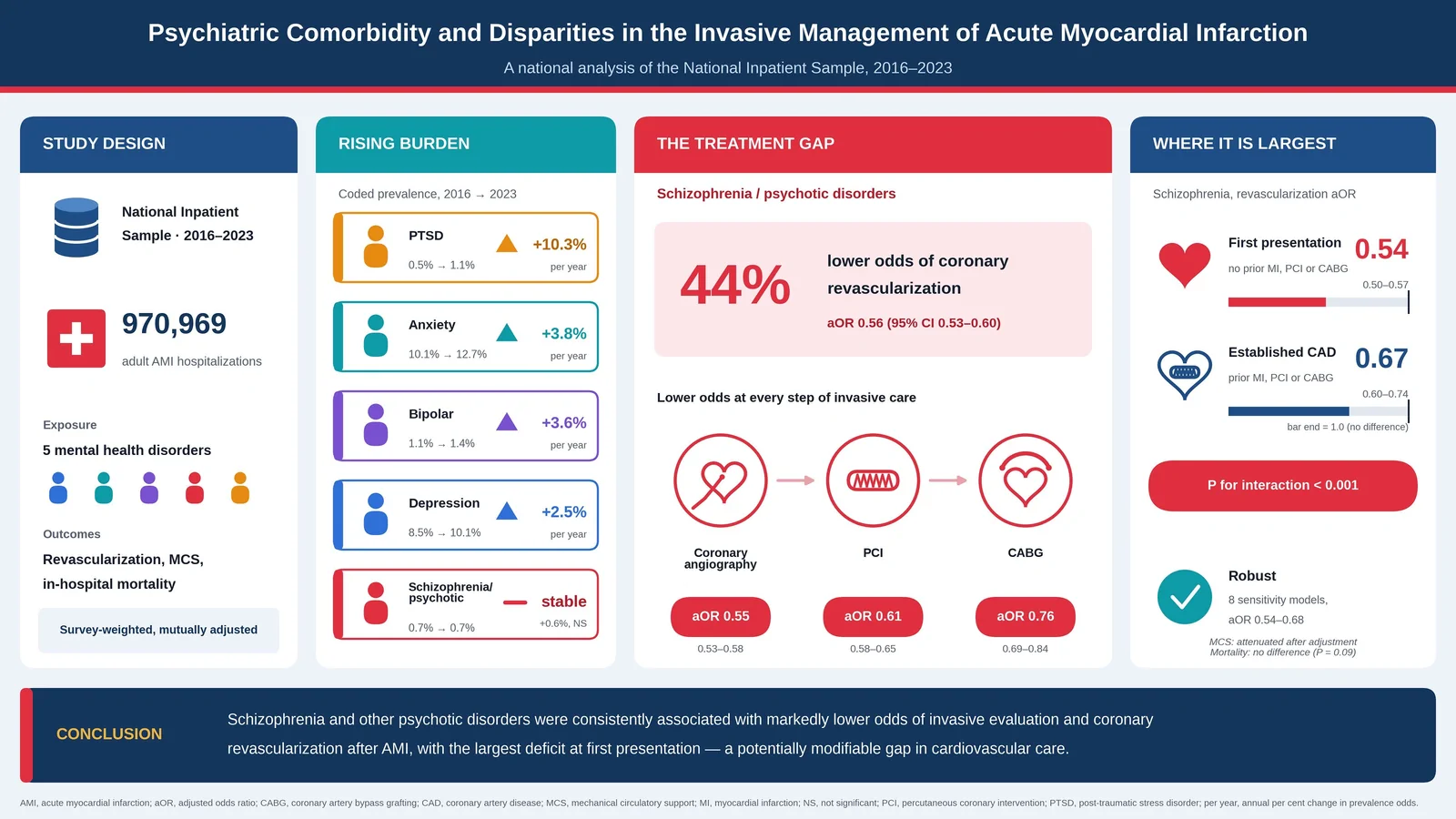

## Introduction

Cardiovascular disease remains the leading cause of death in the USA, and its intersection with mental health is increasingly recognized as a priority for prevention.^1^ The bidirectional relationship between mental health and cardiovascular disease, the ‘mind–heart–body connection’ emphasized in recent American Heart Association (AHA) scientific statements, is now well established.^2–5^ Depression, anxiety, and serious mental illness (SMI) are each associated with an elevated incidence of coronary heart disease and with adverse prognosis after acute coronary events, through a combination of behavioural, autonomic, inflammatory, socioeconomic, and health-system pathways.^6–9^ Beyond incidence, a persistent concern is that patients with mental health disorders (MHDs) receive less intensive evidence-based cardiac care, which may contribute to their excess cardiovascular mortality.^10–14^

A prior analysis of the NIS from 2008 to 2017 documented a rising prevalence of MHD among patients hospitalized for acute myocardial infarction (AMI) and a lower likelihood of coronary revascularization among those with major depression, bipolar disorder, or schizophrenia/psychotic disorders.^15^ Several gaps remain, however. First, that analysis spanned the transition from ICD-9-CM to ICD-10-CM coding and predates the coronavirus disease 2019 (COVID-19) pandemic; contemporary estimates using a single, stable coding system are needed. Second, prior work focused largely on revascularization and mortality. It did not characterize a severity- and treatment-oriented layer, including mechanical circulatory support (MCS), invasive management, complications, and acute organ failure, which is central to modern AMI care. Third, disparities specific to SMI (schizophrenia/psychotic and bipolar disorders), and the extent to which differences in treatment and in-hospital complications may help explain observed mortality associations, warrant exploratory evaluation.^10,16–18^

We therefore conducted a serial cross-sectional analysis of the NIS from 2016 to 2023 to (i) describe the contemporary prevalence and temporal trends of five MHDs among AMI hospitalizations; (ii) quantify the adjusted associations of each MHD with in-hospital mortality, MCS use (overall and by device), and coronary revascularization; and (iii) test the robustness of these associations across expanded mortality, race/region-adjusted, COVID-era, and age-stratified analyses.

## Methods

### Data source and design

This was a serial cross-sectional analysis of the Healthcare Cost and Utilization Project (HCUP) NIS for the years 2016 through 2023. The NIS is the largest publicly available all-payer inpatient database in the USA and approximates a 20% stratified sample of discharges from HCUP participating hospitals. National estimates were generated using the HCUP survey design, incorporating the discharge weight, stratum identifier (NIS_STRATUM), and hospital cluster (HOSP_NIS). The unit of analysis was the hospitalization, not the individual patient. All analyses adhered to AHRQ/HCUP and previously published methodological standards for research using the NIS.^19,20^ Because the entire study period followed the 2012 NIS sampling redesign and used a single coding system (ICD-10-CM/PCS), no trend-weight bridging was required. Reporting follows the STROBE guideline for cross-sectional studies.

### Cohort definition

We included adult hospitalizations (age ≥18 years) with a principal diagnosis of AMI, defined by ICD-10-CM codes I21.x and I22.x. Type 2 myocardial infarction (I21.A) was excluded. Type designations follow the Fourth Universal Definition of Myocardial Infarction.^21^ Cohort assembly is summarized in *Figure 1*.

**Figure 1:**
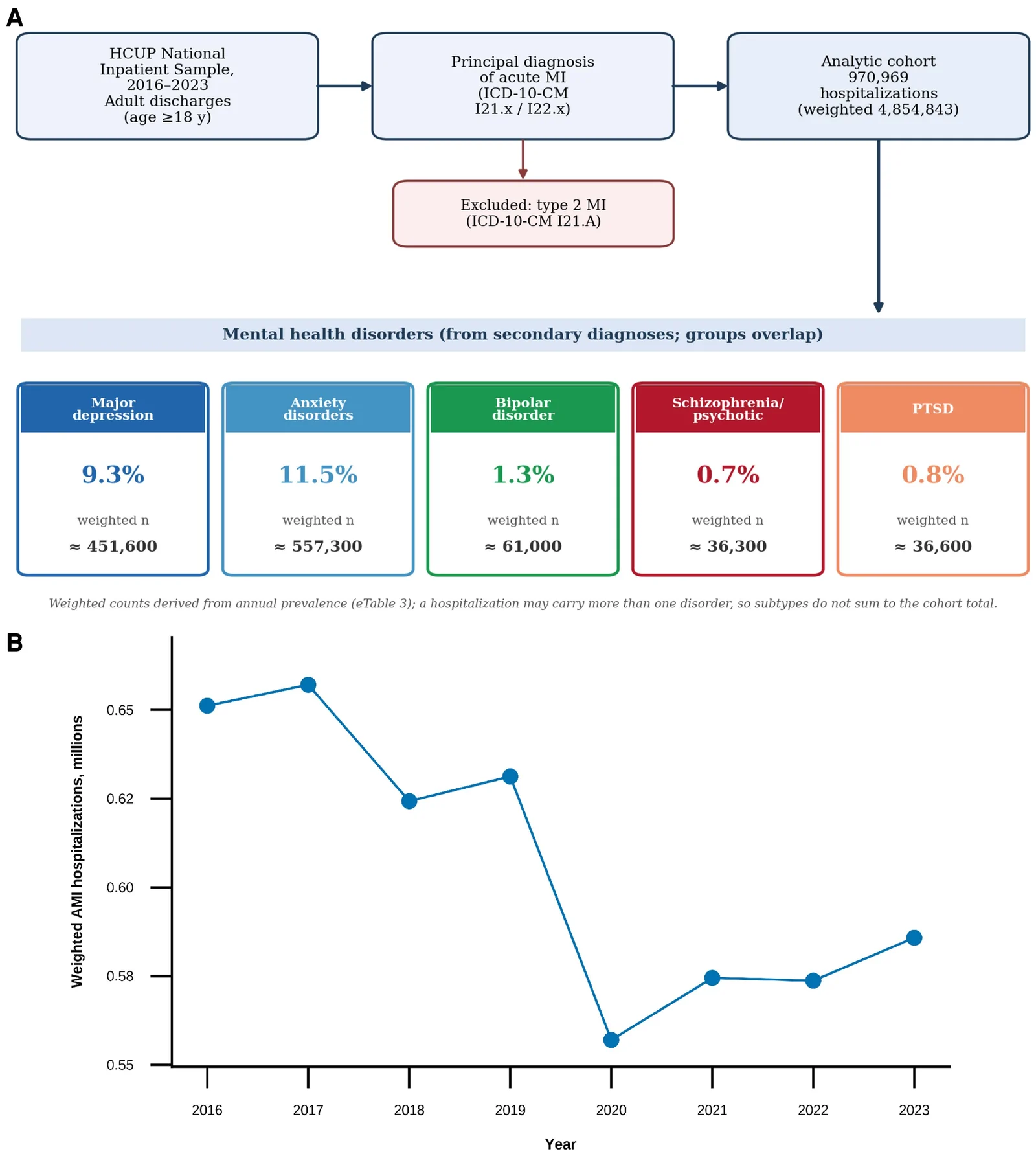
(*A*) Cohort assembly and mental health disorder groups, NIS 2016–23. Weighted subtype counts are derived from annual prevalence (see Supplementary material online, *Table S3*); groups overlap, so subtypes do not sum to the cohort total. (*B*) Weighted national volume of acute myocardial infarction hospitalizations by year, 2016–23, showing the pandemic-related decline in 2020.

### Exposures

The exposures were five MHDs identified using ICD-10-CM codes: major depression, anxiety disorders, bipolar disorder, schizophrenia/other psychotic disorders, and PTSD. Consistent with prior literature,^15,16^ schizophrenia/psychotic and bipolar disorders were together designated SMI. The complete diagnostic codebook is provided in the Supplementary material online.

### Outcomes

The three primary outcomes were (i) in-hospital mortality; (ii) MCS, comprising any device and, individually, intra-aortic balloon pump (IABP), percutaneous left ventricular assist device (pLVAD), and extracorporeal membrane oxygenation (ECMO); and (iii) coronary revascularization [percutaneous coronary intervention (PCI) and coronary artery bypass grafting (CABG)]. Secondary outcomes included coronary angiography, invasive management (any coronary angiography or left heart catheterization code), any complication, acute organ failure (respiratory, hepatic, haematologic, neurologic), mechanical ventilation, acute haemodialysis, palliative-care encounter, do-not-resuscitate (DNR) status, length of stay, and total charges. Procedures and complications were identified by ICD-10-PCS and ICD-10-CM codes (Supplementary material online).

### Covariates and statistical analysis

The main model (2016–23) adjusted for age, sex, primary payer, income quartile, rural residence, hospital teaching status, urban location, and bed size, MI type, and 19 comorbidities (obesity, anaemia, diabetes, hypertension, hyperlipidaemia, atrial fibrillation/flutter, alcohol use disorder, drug use disorder, chronic liver disease, chronic kidney disease, dialysis dependence, pulmonary vascular disease, chronic obstructive pulmonary disease, peripheral arterial disease, dementia, prior MI, prior PCI, prior CABG, and prior stroke), defined using established administrative-data comorbidity approaches.^22^ Covariates were selected *a priori* as factors plausibly influencing both the recording of a psychiatric diagnosis and the decision to pursue an invasive strategy—procedural and bleeding risk, vascular access, competing risk and goals-of-care considerations, insurance status, and hospital capability—rather than as cardiovascular risk factors *per se*; a parsimonious model restricted to this narrower set is reported in Supplementary material online, *Table S16*. Race/ethnicity and hospital region were excluded from the main model because they were suppressed in the NIS 2023 file; a prespecified sensitivity analysis (2016–22) added both variables. This primary model was applied to every outcome, including in-hospital mortality; cardiogenic shock and cardiac arrest were not covariates in the primary model but were examined as outcomes in the full panel (see Supplementary material online, *Table S9*). A single expanded mortality model, applied to in-hospital mortality only, additionally adjusted for disease severity (cardiogenic shock, cardiac arrest), invasive treatment (angiography, PCI, CABG, catheterization, MCS), organ-support procedures, complications, and acute organ-failure indicators. Because several of these variables may lie on the pathway between MHD and mortality, this expanded model was considered exploratory and mediation-informed rather than a formal mediation analysis, and its estimates are susceptible to over-adjustment and collider bias. Further analyses comprised age-stratified models (18–49, 50–75, and ≥76 years); temporal trends in outcomes by the MHD group, with MHD × year interaction terms; and a COVID-era sensitivity analysis (a COVID-era covariate, plus a pre-COVID 2016–19 restriction). Because prior MI, PCI, and CABG identify established coronary disease rather than a first presentation, and alter the revascularization decision itself, two further analyses were performed: the primary models were refitted separately in hospitalizations without any prior MI, PCI, or CABG (incident-event cohort) and in those with established coronary disease, with these three covariates omitted from both; and MHD × prior-coronary-disease interaction terms were added to the full-cohort model, with a joint Wald test across the five disorders (see Supplementary material online, *Tables S14* and *S15*). Subgroup models were fitted on the full survey design using domain estimation rather than by subsetting the data before weighting.

Baseline characteristics were compared using the survey-adjusted Rao–Scott *χ*^2^ test for categorical variables and design-based tests for continuous variables. Survey-weighted logistic regression produced adjusted odds ratio (aORs) with 95% confidence intervals (CIs); the five MHDs were entered simultaneously as indicator variables in each outcome model, so every estimate was mutually adjusted for the co-occurrence of the other four disorders, with the reference for each comprising hospitalizations without that disorder. For temporal trends, calendar year was entered as a continuous variable in survey-weighted logistic regression, and the annual per cent change (APC) in the odds was calculated as (e*^β^* − 1) × 100, where *β* is the calendar-year coefficient; with eight calendar years, a linear (constant log-odds) trend was assumed. Disorder-specific outcome trends were estimated with MHD-by-year interaction terms. Race/ethnicity and hospital region were modelled with an explicit missing category; observations with missing values for other categorical covariates were excluded from the relevant model (complete-case), and survey weights were retained. Because these covariates (primarily primary payer, income quartile, and bed size) have low missingness in the NIS, complete-case exclusions were minimal, and the analytic sample for each model approximated the full cohort. All tests were two-sided with *α* = 0.05. Analyses were performed in R version 4.6.0 (R Foundation for Statistical Computing, Vienna, Austria) with the survey, srvyr, and gtsummary packages. Because the NIS contains de-identified data, the study was exempt from institutional review board review.

## Results

### Cohort and baseline characteristics

A total of 970 969 hospitalizations met the inclusion criteria (weighted, 4 854 843), distributed across the 8 years from 651 174 weighted discharges in 2016 to 585 820 in 2023 (*Figure 1*; *Table 1*). A decline in AMI volume was evident in 2020 (weighted, 557 020) compared with 2019 (631 250), with a partial recovery thereafter.

**Table 1:** Characteristics, treatment patterns, and unadjusted outcomes of acute myocardial infarction hospitalizations by mental health disorder, NIS 2016–23

Characteristic	No MHD	Major depression	Bipolar disorder	Anxiety disorders	Schizo./psychotic	PTSD	P*
Age	67 (13)	66 (13)	59 (12)	65 (13)	62 (13)	60 (12)	<0.001
Sex							<0.001
Male	66.5%	46.2%	50.8%	45.6%	61.7%	69.2%	
Female	33.5%	53.8%	49.2%	54.4%	38.3%	30.8%	
Race/ethnicity							<0.001
White	61.0%	68.2%	65.8%	69.0%	51.1%	63.4%	
Black	10.1%	6.8%	10.3%	6.0%	21.8%	9.1%	
Hispanic	8.0%	6.0%	4.9%	6.0%	8.1%	4.5%	
Asian/PI	2.8%	1.1%	0.7%	1.0%	1.4%	0.9%	
Native American	0.5%	0.5%	0.5%	0.4%	0.4%	0.7%	
Other	2.7%	1.7%	1.9%	1.9%	2.5%	1.7%	
Missing	14.9%	15.8%	16.0%	15.7%	14.7%	19.7%	
Rural residence	17.9%	18.8%	16.8%	18.3%	15.3%	17.9%	0.046
Insurance							<0.001
Medicare	55.4%	62.0%	51.5%	56.6%	59.6%	42.7%	
Medicaid	9.2%	11.0%	23.7%	12.0%	25.1%	17.1%	
Private	27.0%	20.9%	16.2%	24.6%	7.9%	17.0%	
Self-pay	4.9%	2.7%	4.7%	3.6%	3.4%	3.8%	
No charge	0.4%	0.2%	0.4%	0.3%	0.2%	0.2%	
Other	3.1%	3.1%	3.5%	2.7%	3.7%	19.3%	
Income quartile							<0.001
Q1 (low)	30.4%	31.2%	37.7%	31.4%	44.2%	34.6%	
Q2	27.4%	28.7%	28.5%	28.3%	25.5%	29.9%	
Q3	23.5%	23.2%	20.7%	23.2%	18.8%	21.9%	
Q4 (high)	18.6%	16.9%	13.1%	17.1%	11.4%	13.6%	
Hospital bed size							0.047
Small	19.2%	19.3%	20.0%	19.6%	20.0%	18.2%	
Medium	30.3%	29.5%	29.7%	30.1%	30.4%	29.8%	
Large	50.5%	51.2%	50.3%	50.3%	49.6%	52.0%	
Teaching hospital	71.8%	73.0%	73.2%	72.0%	74.4%	75.0%	<0.001
Urban hospital	92.5%	91.7%	92.4%	91.7%	92.3%	92.7%	<0.001
MI type							<0.001
NSTEMI	70.4%	78.3%	75.1%	77.5%	77.0%	74.2%	
STEMI	28.9%	20.9%	24.0%	21.7%	22.1%	25.0%	
Unspecified	0.7%	0.8%	0.9%	0.8%	1.0%	0.7%	
Obesity	20.8%	26.8%	27.2%	25.4%	20.3%	27.5%	<0.001
Anaemia	3.6%	5.1%	4.3%	4.5%	4.8%	3.9%	<0.001
Diabetes	40.8%	45.6%	45.6%	39.2%	45.8%	41.3%	<0.001
Hypertension	81.9%	86.1%	81.8%	84.7%	82.7%	82.5%	<0.001
Hyperlipidaemia	68.4%	75.0%	67.6%	73.2%	59.8%	71.7%	<0.001
Atrial fibrillation/flutter	20.0%	19.3%	13.2%	18.7%	16.3%	15.8%	<0.001
Alcohol use disorder	3.1%	4.7%	8.0%	4.6%	9.0%	8.5%	<0.001
Drug use disorder	3.7%	6.5%	17.6%	7.3%	17.4%	15.0%	<0.001
Chronic liver disease	3.0%	3.0%	4.7%	2.7%	5.5%	3.9%	0.2
CKD	23.7%	26.8%	20.6%	21.9%	24.4%	18.5%	<0.001
Dialysis dependence	3.2%	3.5%	2.5%	2.6%	3.4%	1.6%	0.027
Pulmonary vascular disease	5.3%	6.3%	4.7%	5.8%	5.9%	4.3%	<0.001
COPD	13.6%	22.3%	28.1%	21.8%	27.1%	24.0%	<0.001
PAD	9.9%	11.7%	8.5%	10.8%	8.4%	10.0%	<0.001
Dementia	4.6%	7.9%	5.7%	5.3%	13.1%	3.1%	<0.001
Prior MI	15.6%	18.8%	19.1%	18.0%	17.0%	20.5%	<0.001
Prior PCI	15.4%	16.8%	16.0%	16.0%	12.6%	17.0%	<0.001
Prior CABG	9.3%	9.7%	6.8%	8.5%	6.1%	8.1%	0.001
Prior stroke	7.5%	10.2%	9.9%	8.6%	9.5%	8.7%	<0.001
Any revascularization	59.7%	52.8%	50.9%	54.7%	39.9%	57.4%	<0.001
PCI	51.3%	44.4%	43.2%	45.2%	33.6%	47.5%	<0.001
CABG	8.9%	8.9%	8.1%	10.1%	6.8%	10.4%	<0.001
Coronary angiography	71.7%	69.3%	68.5%	71.2%	54.7%	73.6%	<0.001
Coronary thrombectomy	5.6%	4.3%	4.4%	4.5%	3.5%	4.7%	<0.001
Intravascular ultrasound	6.7%	6.4%	5.8%	6.3%	4.5%	8.4%	<0.001
Fibrinolysis	0.6%	0.5%	0.6%	0.6%	0.5%	0.6%	0.043
Any MCS	5.8%	4.4%	3.9%	4.6%	4.5%	4.2%	<0.001
IABP	4.1%	3.3%	2.9%	3.5%	3.4%	3.3%	<0.001
Percutaneous LVAD (pLVAD)	1.8%	1.2%	1.1%	1.2%	1.2%	0.9%	<0.001
ECMO	0.3%	0.1%	0.1%	0.2%	NR	NR	<0.001
Right heart catheterization	4.1%	3.9%	3.6%	3.7%	3.6%	3.5%	<0.001
Left heart catheterization	65.1%	62.2%	62.6%	64.7%	49.8%	65.3%	<0.001
Invasive mechanical ventilation	6.2%	4.8%	5.4%	4.7%	8.4%	4.8%	<0.001
Non-invasive ventilation	2.8%	3.8%	3.3%	3.7%	4.0%	2.9%	<0.001
Acute haemodialysis	3.5%	3.8%	2.7%	2.9%	3.7%	1.9%	0.001
Respiratory failure	13.2%	14.3%	13.3%	13.9%	17.3%	11.5%	<0.001
Acute kidney injury	20.5%	20.5%	19.3%	18.2%	25.5%	17.5%	<0.001
Hepatic failure	1.8%	1.2%	1.3%	1.1%	1.8%	1.1%	<0.001
Haematologic failure/DIC	4.6%	4.4%	3.9%	4.3%	5.0%	3.9%	0.088
Neurologic failure	3.8%	4.5%	4.8%	3.7%	9.8%	3.5%	<0.001
Cardiac arrest	3.9%	2.6%	2.6%	2.6%	4.0%	3.0%	<0.001
Cardiogenic shock	7.1%	5.5%	5.3%	5.5%	6.7%	4.9%	<0.001
Ischaemic stroke	1.3%	1.3%	1.0%	1.0%	1.5%	0.8%	<0.001
Intracranial haemorrhage	0.2%	0.2%	0.2%	0.1%	0.2%	0.1%	<0.001
Acute limb ischaemia	0.4%	0.4%	0.3%	0.4%	0.3%	0.3%	0.5
Major bleeding	0.9%	0.8%	0.8%	0.7%	1.2%	0.4%	<0.001
Blood transfusion	3.6%	3.9%	3.0%	3.6%	4.1%	2.4%	0.006
Access-site complication	0.2%	0.3%	0.2%	0.2%	0.3%	0.2%	0.034
Any complication	24.0%	23.5%	22.2%	21.2%	29.0%	20.4%	<0.001
Palliative care	3.3%	3.4%	2.2%	3.1%	4.2%	1.8%	0.8
DNR status	8.1%	9.9%	5.8%	8.1%	9.6%	4.9%	<0.001
In-hospital death	5.0%	3.6%	2.8%	3.1%	4.8%	2.3%	<0.001
Discharge disposition							
Routine (home)	65.4%	60.0%	61.6%	63.5%	46.8%	69.5%	
Short-term hospital	7.6%	6.7%	6.5%	6.7%	5.8%	6.3%	
SNF/ICF/facility	9.1%	14.0%	12.8%	11.1%	25.1%	7.9%	
Home health care	11.5%	14.3%	11.6%	13.6%	12.3%	10.4%	
Against medical advice	1.3%	1.4%	4.6%	1.8%	5.1%	3.6%	
Died	5.0%	3.6%	2.8%	3.1%	4.8%	2.3%	
Missing	0.0%	0.0%	0.1%	0.0%	0.1%	0.1%	
Length of stay, days	4.3 (5.5)	4.7 (5.5)	4.5 (5.6)	4.6 (5.0)	6.2 (9.1)	4.4 (5.6)	<0.001
Total charges, $	111 951 (147 280)	103 778 (128 736)	102 439 (130 446)	106 142 (124 271)	112 421 (169 258)	107 590 (122 641)	<0.001

Values are mean (SD) or %. The five MHD subtype columns are not mutually exclusive; a hospitalization may carry more than one MHD. *P is the design-based comparison of No MHD vs. Any MHD only (Rao–Scott χ^2^ for categorical, Kruskal–Wallis for continuous variables); it is not an omnibus test across the displayed subtype columns. Subtype-specific descriptive comparisons are provided in the supplement. NR, not reportable: the underlying cell contains 10 or fewer unweighted discharges, so the corresponding percentage is suppressed in accordance with the HCUP Data Use Agreement (see Supplementary material online, *Table S17*).

Baseline characteristics differed markedly across MHD groups (*Table 1*). Patients with schizophrenia/psychotic disorders and bipolar disorder were younger (mean age 62 and 59 years, respectively) than those without MHD (67 years) and more frequently insured by Medicaid (25.1 and 23.7 vs. 9.2%) and in the lowest income quartile (44.2 and 37.7 vs. 30.4%). PTSD hospitalizations were predominantly male (69.2%), whereas depression and anxiety hospitalizations were more often female (53.8 and 54.4%). Non-ST-elevation MI predominated in all MHD groups (schizophrenia/psychotic, 77.0%; depression, 78.3%) relative to those without MHD (70.4%). Patients with schizophrenia/psychotic disorders had a longer mean length of stay (6.2 vs. 4.3 days). They were more often discharged to a skilled nursing or intermediate-care facility (25.1 vs. 9.1%) or against medical advice (5.1 vs. 1.3%).

### Prevalence and temporal trends

Across 2016–23, anxiety disorders (10.1→12.7%) and major depression (8.5→10.1%) were the most prevalent MHD, followed by bipolar disorder (1.1→1.4%), schizophrenia/psychotic disorders (0.7→0.7%), and PTSD (0.5→1.1%) (*Table 3*; *Figure 2*). The prevalence rose significantly for anxiety (APC +3.8%; 95% CI, 3.4–4.2), major depression (APC +2.5%; 95% CI, 2.0–3.0), bipolar disorder (APC +3.6%; 95% CI, 2.8–4.5), and, most steeply, PTSD (APC +10.3%; 95% CI, 9.0–11.6; all *P* < 0.001), whereas schizophrenia/psychotic disorders were stable (APC +0.6%; 95% CI, −0.5–1.7; *P* = 0.316). These inpatient increases parallel the rising population-level prevalence of depression and anxiety reported in national surveys.^23–25^ Because the five disorders overlap, disorder-specific prevalence rather than an aggregate any-MHD estimate is reported (*Table 3*).

**Figure 2:**
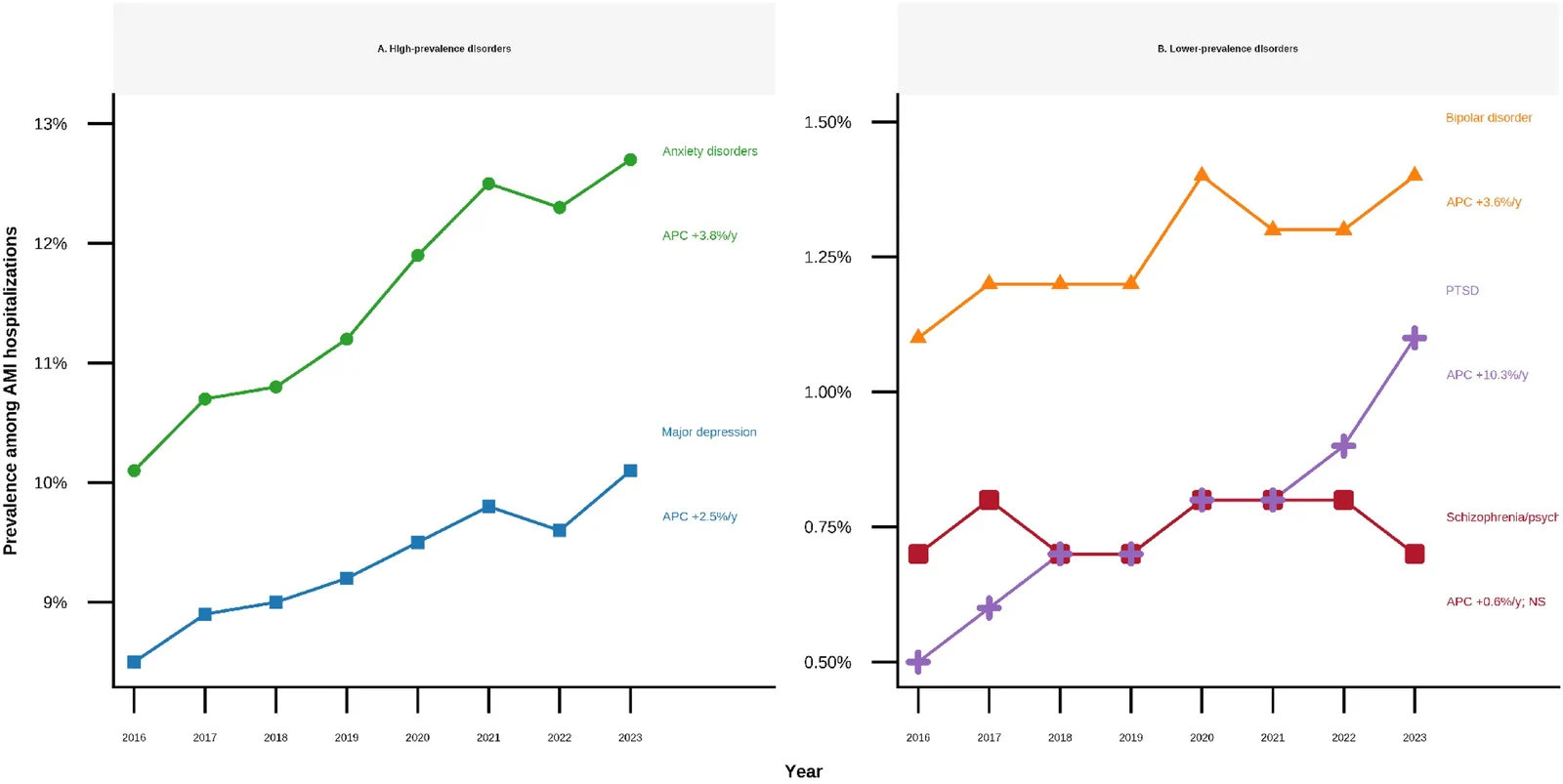
Prevalence of mental health disorder among acute myocardial infarction hospitalizations, 2016–23, plotted on separate scales for high-prevalence (*A*) and lower-prevalence (*B*) disorders. APC, annual per cent change; NS, not significant.

To gauge background coding drift, we compared the MHD trends with secular coding comparator conditions (see Supplementary material online, *Table S2*). Documented hyperlipidaemia rose over the same interval (APC +4.7%; 95% CI, 4.3–5.2) and anaemia rose modestly (APC +2.2%; 95% CI, 1.6–2.8), while peripheral arterial disease declined (APC −2.6%; 95% CI, −3.1 to −2.2). Because the magnitude of the depression, anxiety, and bipolar disorder increases was comparable to that of the hyperlipidaemia comparator, these more modest MHD rises should be interpreted cautiously as potentially reflecting, in part, increasing coding intensity, a phenomenon documented for psychiatric diagnoses in administrative data.^26–28^ The PTSD increase (APC +10.3%) exceeded that of the selected coding comparators, suggesting it may not be fully attributable to the degree of coding drift captured by those conditions.

### Primary outcomes: revascularization and mechanical support

After multivariable adjustment (*Table 2*; *Figure 3*), schizophrenia/psychotic disorders were associated with the lowest odds of coronary revascularization (aOR, 0.56; 95% CI, 0.53–0.60; *P* < 0.001) and of PCI (aOR, 0.61; 95% CI, 0.58–0.65; *P* < 0.001). Bipolar disorder was also associated with substantially lower revascularization (aOR, 0.81; 95% CI, 0.78–0.85; *P* < 0.001) and PCI (aOR, 0.85; 95% CI, 0.82–0.89; *P* < 0.001). The reductions associated with major depression (revascularization aOR, 0.94; 95% CI, 0.92–0.95; *P* < 0.001) and PTSD (aOR, 0.93; 95% CI, 0.89–0.98; *P* = 0.009) were smaller in magnitude, and anxiety disorders were not significantly associated with revascularization (aOR, 0.98; 95% CI, 0.97–1.00; *P* = 0.06). A parallel gradient was observed for invasive management, which was least likely among hospitalizations with schizophrenia/psychotic disorders (aOR, 0.52; 95% CI, 0.49–0.55; *P* < 0.001) and bipolar disorder (aOR, 0.85; 95% CI, 0.81–0.89; *P* < 0.001), and for coronary angiography (schizophrenia/psychotic aOR, 0.55; 95% CI, 0.53–0.58; *P* < 0.001). Anxiety disorders were an exception among the procedures, being associated with higher odds of CABG (aOR, 1.22; 95% CI, 1.19–1.25; *P* < 0.001) despite near-neutral odds of angiography and revascularization.

**Figure 3:**
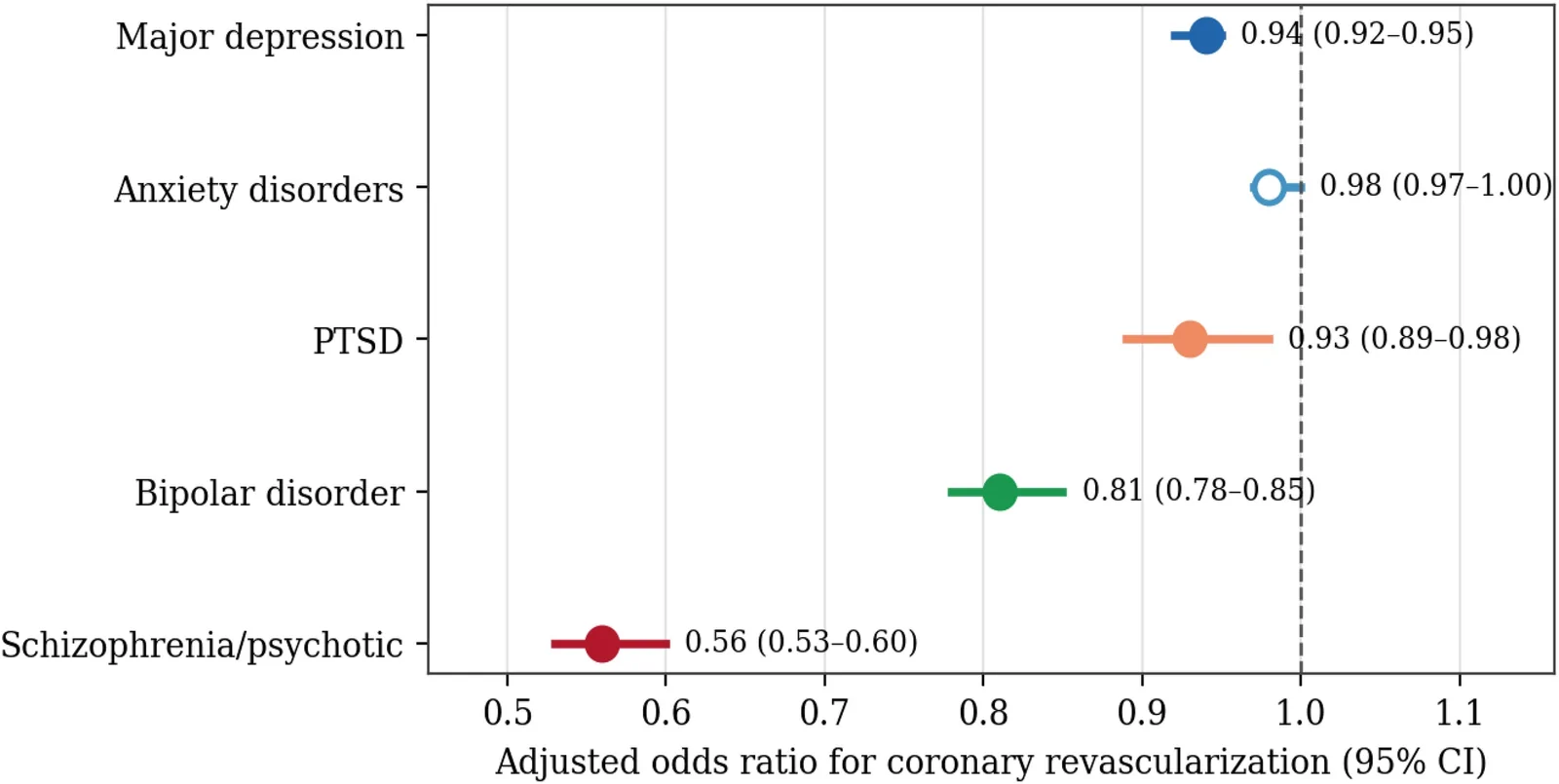
Adjusted odds of coronary revascularization by mental health disorder (main model, 2016–23). Filled markers indicate 95% confidence intervals that do not include 1.0.

**Table 2:** Adjusted odds of the principal in-hospital outcomes for each mental health disorder vs. no such disorder (main model, 2016–23)

Outcome	Major depression	Anxiety disorders	Bipolar disorder	Schizophrenia/psychotic	PTSD
Coronary angiography	*0.99 (0.97–1.01): P* = 0*.42*	1.03 (1.01–1.05)*: P* = 0*.002*	0.86 (0.83–0.90)*: P* *<* 0*.001*	0.55 (0.53–0.58)*: P* *<* 0*.001*	*1.01 (0.95–1.06): P* = 0*.85*
Invasive management	0.98 (0.96–0.99)*: P* = 0*.01*	*1.02 (1.00–1.04): P* = 0*.05*	0.85 (0.81–0.89)*: P* *<* 0*.001*	0.52 (0.49–0.55)*: P* *<* 0*.001*	*1.00 (0.95–1.07): P* = 0*.89*
Any revascularization	0.94 (0.92–0.95)*: P* *<* 0*.001*	*0.98 (0.97–1.00): P* = 0*.06*	0.81 (0.78–0.85)*: P* *<* 0*.001*	0.56 (0.53–0.60)*: P* *<* 0*.001*	0.93 (0.89–0.98)*: P* = 0*.009*
PCI	0.97 (0.95–0.98)*: P* *<* 0*.001*	0.92 (0.90–0.93)*: P* *<* 0*.001*	0.85 (0.82–0.89)*: P* *<* 0*.001*	0.61 (0.58–0.65)*: P* *<* 0*.001*	0.93 (0.88–0.98)*: P* = 0*.006*
CABG	0.93 (0.91–0.96)*: P* *<* 0*.001*	1.22 (1.19–1.25)*: P* *<* 0*.001*	0.88 (0.82–0.94)*: P* *<* 0*.001*	0.76 (0.69–0.84)*: P* *<* 0*.001*	*1.00 (0.93–1.09): P* = 0*.94*
Any MCS	0.91 (0.88–0.94)*: P* *<* 0*.001*	*1.02 (0.99–1.05): P* = 0*.26*	0.78 (0.71–0.86)*: P* *<* 0*.001*	*0.89 (0.79–1.00): P* = 0*.05*	0.83 (0.73–0.94)*: P* = 0*.002*
IABP	0.94 (0.90–0.97)*: P* = 0*.002*	1.05 (1.01–1.09)*: P* = 0*.02*	0.80 (0.72–0.90)*: P* *<* 0*.001*	*0.94 (0.83–1.08): P* = 0*.41*	*0.90 (0.79–1.02): P* = 0*.11*
Percutaneous LVAD	0.86 (0.80–0.92)*: P* *<* 0*.001*	*0.95 (0.89–1.01): P* = 0*.09*	0.76 (0.64–0.92)*: P* = 0*.003*	*0.81 (0.65–1.01): P* = 0*.07*	0.61 (0.47–0.79)*: P* *<* 0*.001*
ECMO	*0.84 (0.70–1.01): P* = 0*.06*	0.81 (0.68–0.95)*: P* = 0*.01*	*0.66 (0.41–1.08): P* = 0*.10*	NR	*NR*
In-hospital mortality	0.86 (0.82–0.90)*: P* *<* 0*.001*	0.81 (0.78–0.84)*: P* *<* 0*.001*	0.81 (0.72–0.91)*: P* *<* 0*.001*	*1.11 (0.98–1.26): P* = 0*.09*	0.69 (0.58–0.81)*: P* *<* 0*.001*

Each cell shows the adjusted odds ratio (95% CI) and the *P*-value for hospitalizations without the respective disorder, from survey-weighted logistic regression (main model, 2016–23). Grey, italicized cells denote 95% CIs including 1.0. MCS, mechanical circulatory support; PCI, percutaneous coronary intervention; CABG, coronary artery bypass grafting; LVAD, left ventricular assist device; IABP, intra-aortic balloon pump; ECMO, extracorporeal membrane oxygenation. Full model covariates and the complete outcome panel are in Supplementary material online, *Table S4*. NR, not reportable: the underlying cell contains 10 or fewer unweighted discharges and is therefore suppressed in accordance with the HCUP Data Use Agreement (see Supplementary material online, *Table S17*).

**Table 3:** Temporal trend (annual per cent change in prevalence odds) of each mental health disorder among acute myocardial infarction hospitalizations, 2016–23

Mental health disorder	APC (95% CI)	*P* for trend
Major depression	+2.5 (2.0, 3.0)	<0.001
Anxiety disorders	+3.8 (3.4, 4.2)	<0.001
Bipolar disorder	+3.6 (2.8, 4.5)	<0.001
Schizophrenia/psychotic disorders	+0.6 (−0.5, 1.7)	0.316
PTSD	+10.3 (9.0, 11.6)	<0.001

APC, annual per cent change in the odds of the disorder per calendar year, from survey-weighted logistic regression. Year-by-year prevalence is provided in Supplementary material online, *Table S3*.

For MCS, the pattern was device-dependent. Any-MCS use was lower with bipolar disorder (aOR, 0.78; 95% CI, 0.71–0.86; *P* < 0.001), major depression (aOR, 0.91; 95% CI, 0.88–0.94; *P* < 0.001), PTSD (aOR, 0.83; 95% CI, 0.73–0.94; *P* = 0.002), and, at the margin, schizophrenia/psychotic disorders (aOR, 0.89; 95% CI, 0.79–1.00; *P* = 0.05), but not anxiety (aOR, 1.02; 95% CI, 0.99–1.05; *P* = 0.26). For schizophrenia/psychotic disorders, no device-level deficit reached significance after adjustment: pLVAD (aOR, 0.81; 95% CI, 0.65–1.01; *P* = 0.07) and IABP (aOR, 0.94; 95% CI, 0.83–1.08; *P* = 0.41). Extracorporeal membrane oxygenation use in this group involved too few events for stable or reportable estimation and is not presented, in keeping with HCUP cell-size reporting requirements. Bipolar disorder showed a reduction for pLVAD (aOR, 0.76; 95% CI, 0.64–0.92; *P* = 0.003) but not ECMO (aOR, 0.66; 95% CI, 0.41–1.08; *P* = 0.10), as did PTSD for pLVAD (aOR, 0.61; 95% CI, 0.47–0.79; *P* < 0.001). Across the care cascade for schizophrenia/psychotic disorders (*Figure 4*), the adjusted deficit was present and significant from coronary angiography and invasive management through revascularization and PCI, and attenuated to non-significance for MCS and in-hospital mortality.

**Figure 4:**
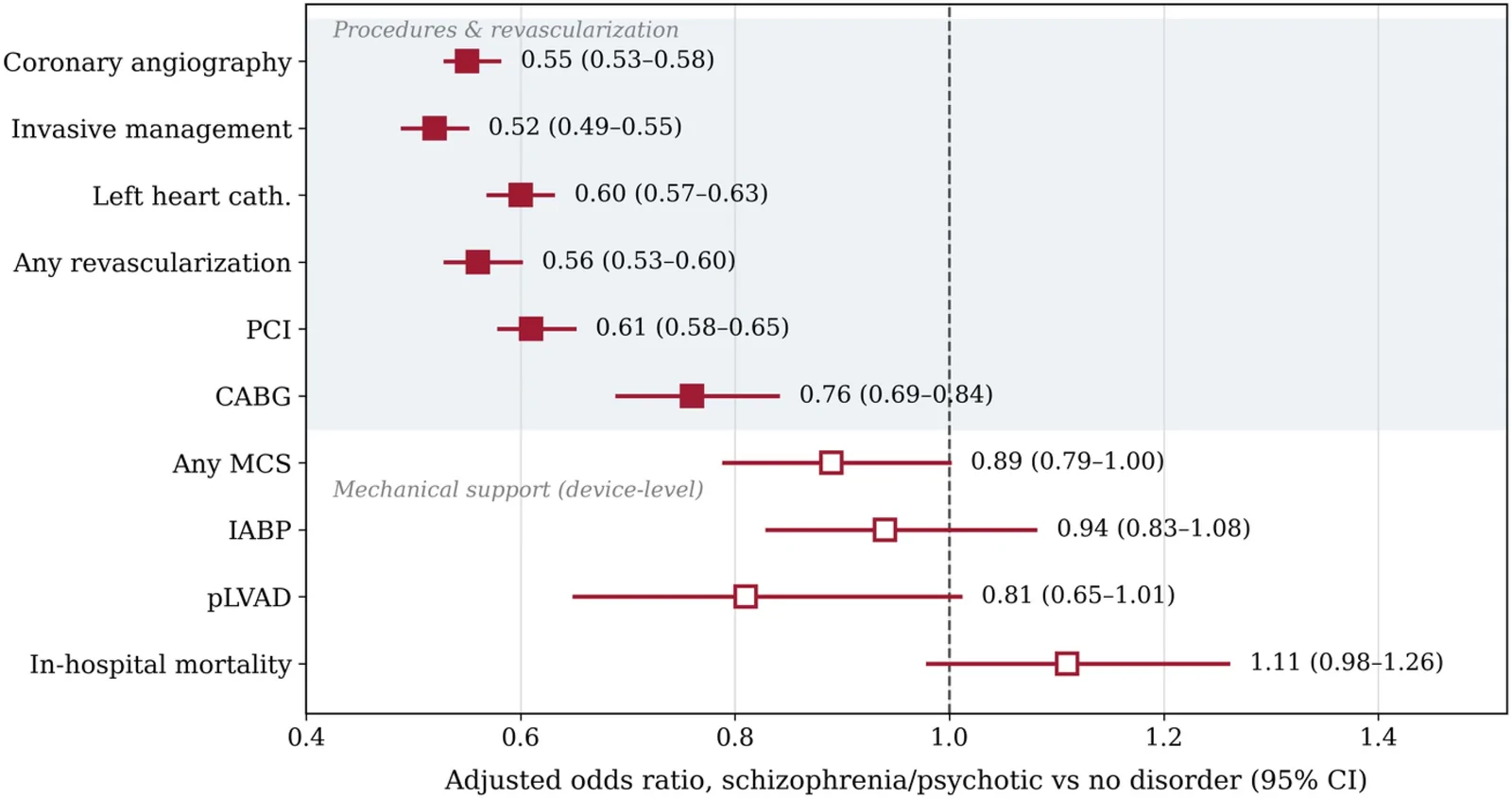
Schizophrenia/psychotic disorders across the acute myocardial infarction care cascade (main model). Adjusted odds ratios (95% confidence interval) vs. hospitalizations without the disorder (filled markers, confidence interval excludes 1.0). The deficit is significant for diagnostic and revascularization procedures, whereas any mechanical circulatory support, intra-aortic balloon pump, percutaneous left ventricular assist device, and in-hospital mortality cross 1.0. Extracorporeal membrane oxygenation is not shown because the number of events was too small for stable or reportable estimation.

### In-hospital mortality

In the baseline-adjusted model (*Table 2*), major depression (aOR, 0.86; 95% CI, 0.82–0.90), anxiety disorders (aOR, 0.81; 95% CI, 0.78–0.84), PTSD (aOR, 0.69; 95% CI, 0.58–0.81), and bipolar disorder (aOR, 0.81; 95% CI, 0.72–0.91) were each associated with lower in-hospital mortality (all *P* < 0.001). In contrast, schizophrenia/psychotic disorders showed no significant difference in adjusted mortality (aOR, 1.11; 95% CI, 0.98–1.26; *P* = 0.09).

In an expanded model additionally adjusted for treatment, complications, and acute organ failure (see Supplementary material online, *Table S8*), the point estimate for schizophrenia/psychotic disorders shifted downward to an aOR of 0.86 (95% CI, 0.73–1.01; *P* = 0.07), a directional change that did not reach statistical significance.

### Secondary outcomes and illness severity

Hospitalizations with schizophrenia/psychotic disorders had higher adjusted odds of several severity and process markers, including any complication (aOR, 1.25; 95% CI, 1.18–1.32; *P* < 0.001), invasive mechanical ventilation (aOR, 1.37; 95% CI, 1.24–1.51; *P* < 0.001), acute kidney injury (aOR, 1.27; 95% CI, 1.19–1.35; *P* < 0.001), acute respiratory organ failure (aOR, 1.18; 95% CI, 1.10–1.27; *P* < 0.001), cardiac arrest (aOR, 1.16; 95% CI, 1.02–1.32; *P* = 0.02), major bleeding (aOR, 1.39; 95% CI, 1.11–1.73; *P* = 0.004), palliative-care encounter (aOR, 1.27; 95% CI, 1.12–1.45; *P* < 0.001), and DNR status (aOR, 1.35; 95% CI, 1.22–1.49; *P* < 0.001) (see Supplementary material online, *Table S9*). Depression, anxiety, and PTSD were generally associated with lower or unchanged odds of these complications, although DNR status was more frequent with depression (aOR, 1.27; 95% CI, 1.23–1.30; *P* < 0.001) and bipolar disorder (aOR, 1.32; 95% CI, 1.21–1.44; *P* < 0.001). Lower invasive treatment use thus coexisted with a greater documented burden of several in-hospital complications and organ-failure indicators; because the NIS does not time-stamp diagnoses, their temporal relationship to treatment decisions cannot be determined.

### Sensitivity, COVID-era, and age-stratified analyses

The schizophrenia revascularization gap was robust. In the sensitivity model additionally adjusted for race/ethnicity and hospital region (2016–22; Supplementary material online, *Table S7*), the association was essentially unchanged (revascularization aOR, 0.59; 95% CI, 0.56–0.63; *P* < 0.001; PCI aOR, 0.63; 95% CI, 0.59–0.67; *P* < 0.001), and adjusted mortality remained non-significant (aOR, 1.10; 95% CI, 0.96–1.25; *P* = 0.17). In the COVID-era model (see Supplementary material online, *Table S10*), the schizophrenia revascularization estimate was essentially unchanged from the main model (aOR, 0.56; 95% CI, 0.53–0.60; *P* < 0.001) and persisted in the pre-COVID 2016–19 restriction (aOR, 0.57; 95% CI, 0.52–0.61; *P* < 0.001). Age stratification (see Supplementary material online, *Table S6*) showed a consistent deficit in every age band: 18–49 years (aOR, 0.58; 95% CI, 0.50–0.67; *P* < 0.001), 50–75 years (aOR, 0.54; 95% CI, 0.51–0.57; *P* < 0.001), and ≥76 years (aOR, 0.68; 95% CI, 0.58–0.79; *P* < 0.001). These robustness analyses are summarized in *Figure 5*.

**Figure 5:**
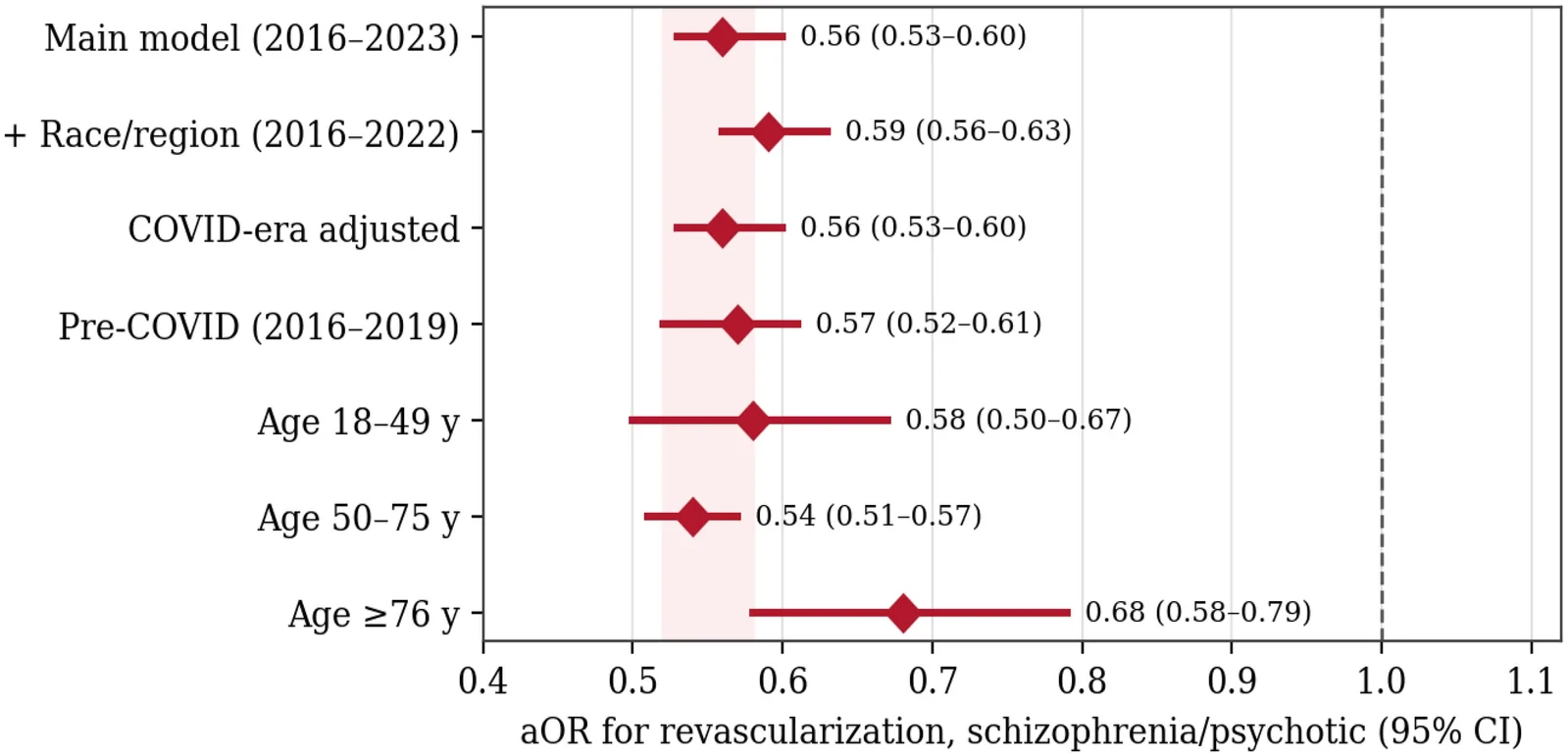
Robustness of the schizophrenia/psychotic revascularization deficit across the main, race/region-adjusted, COVID-era, pre-COVID, and age-stratified models. Sources: *Table 2*, Supplementary material online, *Tables S6, S7*, and *S10*.

Because prior myocardial infarction, PCI, or CABG identifies established coronary disease rather than a first presentation, and alters the revascularization decision itself, the primary models were refitted separately by prior coronary disease status (see Supplementary material online, *Table S14*). Among 669 715 hospitalizations without prior MI, PCI, or CABG, the schizophrenia revascularization deficit was larger than in the full cohort (aOR, 0.54; 95% CI, 0.50–0.57; *P* < 0.001), as was the deficit in invasive management (aOR, 0.50; 95% CI, 0.47–0.53; *P* < 0.001); among 282 793 hospitalizations with established coronary disease it was attenuated but persisted (revascularization aOR, 0.67; 95% CI, 0.60–0.74; *P* < 0.001). In the full cohort, the schizophrenia × prior-coronary-disease interaction was significant for revascularization (ratio of aORs, 1.29; 95% CI, 1.14–1.46; *P* < 0.001), with a significant joint test across the five disorders for revascularization and PCI (both *P* < 0.001) but not for invasive management (*P* = 0.05) or angiography (*P* = 0.07) (see Supplementary material online, *Table S15*). Depression and anxiety showed the same direction of modification; bipolar disorder did not. PTSD was the one disorder whose revascularization association did not persist in the incident-event cohort (aOR, 0.96; 95% CI, 0.90–1.03; *P* = 0.23), and adjusted mortality for schizophrenia/psychotic disorders remained non-significant in both strata. A parsimonious model restricted to covariates plausibly affecting the invasive-treatment decision produced closely similar procedural estimates (schizophrenia revascularization aOR, 0.54; 95% CI, 0.51–0.57), although the schizophrenia mortality estimate was sensitive to specification (aOR, 1.20; 95% CI, 1.06–1.36) (see Supplementary material online, *Table S16*).

### Outcome trends by mental health disorder group

Revascularization rose over the study period overall (APC +2.0%; 95% CI, 1.6–2.5) and among hospitalizations without MHD (APC +2.0%; 95% CI, 1.5–2.4), major depression (APC +3.2%; 95% CI, 2.5–4.0), and anxiety disorders (APC +2.7%; 95% CI, 2.0–3.4) (see Supplementary material online, *Table S5*). In schizophrenia/psychotic disorders, however, revascularization did not increase significantly (APC +2.0%; 95% CI, −0.2–4.3; *P* = 0.071) and remained the lowest among groups across years (39.7% in 2016–41.1% in 2023); PCI followed the same pattern. Mental health disorder × year interaction terms were significant for revascularization trajectories in depression, anxiety, bipolar, and PTSD groups, indicating divergent temporal patterns across MHD categories. In-hospital mortality trends were flat across all groups (overall APC +0.2%; 95% CI, −0.2–0.7; *P* = 0.264). Overall MCS use rose modestly over the study period (any-MCS composite APC +2.6%; 95% CI, 2.0–3.2); device-specific trajectories by MHD group, which were unstable within small subgroups, are shown in Supplementary material online, *Table S5*.

## Discussion

Three findings emerge from this analysis of 970 969 AMI hospitalizations (weighted, 4 854 843) between 2016 and 2023. The coded prevalence of every MHD except schizophrenia/psychotic disorders increased across the period, most steeply for PTSD. After multivariable adjustment, SMI—particularly schizophrenia/psychotic disorders—was associated with lower odds of coronary angiography, invasive management, revascularization, and PCI; associations with MCS were directionally similar but did not reach significance after adjustment. The revascularization deficit was largest among patients presenting without prior coronary disease and narrowed among those with established coronary disease. Depression, anxiety, PTSD, and bipolar disorder were associated with lower adjusted in-hospital mortality. In contrast, schizophrenia/psychotic disorders showed no mortality difference in the primary model and only a downward, non-significant shift after conditioning on treatment and complications.

### Rising burden of mental illness among patients with acute myocardial infarction

Coded prevalence increased for major depression (APC +2.5%; 95% CI, 2.0–3.0), anxiety disorders (+3.8%; 3.4–4.2), bipolar disorder (+3.6%; 2.8–4.5), and PTSD (+10.3%; 9.0–11.6), while schizophrenia/psychotic disorders remained stable (+0.6%; −0.5–1.7). The direction and the stability of schizophrenia reproduce the earlier NIS analysis of 2008–17 in an independent, single-coding-system period,^15^ and parallel the rising population prevalence of depression and anxiety reported in national survey data.^23–25^ These increases should not be read as entirely biological: the secular coding comparator conditions rose concurrently, the accuracy of routinely coded psychiatric diagnoses is imperfect,^26^ and secular changes in psychiatric coding have been documented previously.^27,28^ The depression, anxiety, and bipolar disorder increases are therefore of a magnitude compatible with coding drift. In contrast, the PTSD increase exceeded that of the selected comparators and may not be fully attributable to the coding drift they capture.

### Undertreatment in serious mental illness

Schizophrenia/psychotic disorders carried the lowest adjusted odds of coronary angiography (aOR, 0.55; 95% CI, 0.53–0.58), invasive management (0.52; 0.49–0.55), revascularization (0.56; 0.53–0.60), PCI (0.61; 0.58–0.65), and CABG (0.76; 0.69–0.84); bipolar disorder showed intermediate deficits (revascularization 0.81; 0.78–0.85). These estimates agree with US and international data reporting lower use of invasive cardiac procedures and revascularization after AMI in SMI,^11,13,16,17^ including the specific findings of Mohamed *et al.*^16^ in US hospitalizations and Fleetwood *et al.*^17^ for post-MI revascularization, and with earlier reports of process-of-care disparities in patients with mental disorders.^12,29,30^ The magnitude of the association differed considerably across diagnostic categories and was greatest for schizophrenia/psychotic disorders. Mechanisms proposed for this gap, and not resolvable in administrative data, include diagnostic overshadowing, impaired symptom reporting, capacity and consent barriers, and fragmented access to specialist cardiac services.^10^ Features consistent with these barriers were present in the cohort: against-medical-advice discharge (5.1 vs. 1.3%) and Medicaid insurance (25.1 vs. 9.2%) were both more frequent than in hospitalizations without any MHD (*Table 1*). These in-hospital findings sit at the end of a longer chain: inadequate screening and primary prevention in a population already at elevated cardiovascular risk from lifestyle factors and from the cardiometabolic effects of antipsychotic treatment precede the treatment gap described here.^31,32^

The deficit was concentrated at first presentation. Among hospitalizations without prior myocardial infarction, PCI, or CABG, the schizophrenia revascularization estimate was lower than in the full cohort (0.54; 0.50–0.57), and it attenuated among patients with established coronary disease (0.67; 0.60–0.74), with a significant interaction. One interpretation is that the decision to proceed invasively is most discretionary at the index event, when coronary anatomy is unknown and no established cardiology relationship exists, and that this is where diagnostic overshadowing and assumptions about capacity and adherence have the greatest scope to operate. Depression and anxiety showed the same pattern; bipolar disorder did not, its deficit being present irrespective of prior coronary disease. Because the interaction is multiplicative and baseline revascularization differs between the two strata, this difference is in relative rather than absolute terms.

### Device-specific associations with mechanical circulatory support

In the fully adjusted model (which included alcohol- and drug-use disorders), no device-level reduction in schizophrenia/psychotic disorders reached significance: any MCS (0.89; 0.79–1.00), pLVAD (0.81; 0.65–1.01), and IABP (0.94; 0.83–1.08). Extracorporeal membrane oxygenation use was too infrequent in this group for stable or reportable estimation and is not presented. A directionally similar device pattern was seen for bipolar disorder and, for pLVAD, PTSD. Prior disparity research has examined revascularization and generic procedure use;^13,16^ to our knowledge, device-level differences in MCS have not previously been characterized in SMI. These device-level analyses rest on small numbers of events, are considerably weaker than the robust angiography and revascularization deficits, and should be regarded as exploratory and in need of external replication.

### In-hospital mortality and potential explanatory pathways

Major depression (0.86; 0.82–0.90), anxiety disorders (0.81; 0.78–0.84), bipolar disorder (0.81; 0.72–0.91), and PTSD (0.69; 0.58–0.81) were associated with lower adjusted in-hospital mortality, whereas schizophrenia/psychotic disorders were not (1.11; 0.98–1.26). A comparable in-hospital mortality pattern was reported in the 2008–17 NIS analysis and attributed in part to the younger age of patients with recorded MHD,^15^ and reliance on secondary-diagnosis coding is a recognized source of bias in mortality associations after AMI.^33^ Because these disorders were identified only from secondary diagnosis fields, differential ascertainment is a specific concern: hospitalizations ending in early death or abbreviated stays may have fewer secondary conditions coded, which alone could generate an artifactual inverse association between recorded MHD and in-hospital mortality. The lower-mortality signal is thus more plausibly explained by case mix and ascertainment—patients with these disorders were younger and more often presented with non-ST-elevation infarction (*Table 1*)—than by a protective effect, particularly because depression and PTSD carry adverse longer-term cardiovascular prognosis^4,6,7,34–37^ and psychiatric comorbidity increases readmission after AMI.^38,39^ For schizophrenia specifically, the point estimate moved from 1.11 in the primary model to 0.86 (0.73–1.01; *P* = 0.07) after additional adjustment for treatment, complications, and acute organ failure, a downward shift that was not significant. Because these variables lie on the causal path between the exposure and death, the directional shift is compatible with the possibility that treatment and in-hospital complications contribute to the observed mortality association, but it cannot establish mediation; a comparable pattern was reported by Kugathasan *et al.*,^18^ who linked lower receipt of guideline secondary prevention after MI to excess mortality in schizophrenia, yet the non-significant estimate and the potential for over-adjustment and collider bias make the expanded-model result exploratory rather than confirmatory. The absence of an in-hospital mortality signal should not be read as an absence of excess risk: meta-analytic data show higher 30-day, 1-year, and overall post–acute coronary syndrome mortality in SMI alongside lower receipt of revascularization and of cardioprotective medication,^40^ and disparities in cardiovascular screening and treatment are documented across mental disorders internationally.^31^

### Robustness and generalizability

The schizophrenia revascularization deficit was stable across the sensitivity model adding race/ethnicity and hospital region (0.59; 0.56–0.63), the COVID-era-adjusted model (0.56; 0.53–0.60), the pre-COVID restriction (0.57; 0.52–0.61), and every age stratum (0.54–0.68) (*Figure 5*). Its persistence argues against explanation by pandemic-related disruption of invasive services, by residual confounding from the race and region variables suppressed in the 2023 file, or by age. A quantitatively similar revascularization deficit after AMI has been reported for another chronic, stigma-associated condition, autoimmune rheumatic disease,^41^ which is compatible with shared health-system drivers, though this comparison is hypothesis-generating.

### Clinical relevance

Because the treatment gap was concentrated in SMI, a younger, disproportionately Medicaid-insured group presenting predominantly with non-ST-elevation infarction, it is most actionable at the index admission, where the decision to perform angiography and revascularization is made. The factors known to shape that decision are largely structural rather than clinical: limited collaboration between psychiatry and cardiology, the presumption that patients with SMI lack capacity to consent, and the expectation that they will not adhere to treatment after infarction—an expectation not borne out when patients receiving antipsychotic treatment are adequately supported. Integrated cardiac–psychiatric care is the framework advanced in current scientific statements for addressing this population,^2,3^ and the 2025 ESC clinical consensus statement sets out operational pathways for systematic mental-health screening and stepped care within cardiovascular services;^42^ structured cardiac rehabilitation improves psychological and prognostic outcomes after cardiovascular events.^43^ The data presented here suggest that integration may be particularly valuable at the point of invasive evaluation and revascularization decision-making, and at first presentation in particular.

### Limitations

Several limitations warrant emphasis. First, MHD, comorbidities, procedures, and complications were ascertained from administrative ICD-10 codes; the accuracy of routinely coded psychiatric diagnoses is imperfect and varies by disorder and coding algorithm (positive predictive value on the order of 76% in one systematic review), and misclassification may bias associations.^26^ Our secular coding comparators demonstrated measurable background coding drift, reinforcing cautious interpretation of the more modest prevalence trends.^27,28^ Because MHDs were ascertained from secondary diagnosis fields, differential ascertainment may also bias mortality associations, as discussed above. Second, the NIS unit of analysis is the hospitalization, not the patient, so individuals with multiple admissions may be counted more than once, and no post-discharge or long-term outcomes are available. Prescribing of secondary prevention medication, a plausible contributor to post-discharge outcome differences in SMI, cannot be assessed in the NIS; linked pharmacy data would be required to address it.^40,44^ Third, despite extensive multivariable adjustment, residual and unmeasured confounding (e.g. infarct severity beyond MI type, coronary anatomy, medication use, do-not-intervene preferences, and functional status) is likely; the expanded mortality model additionally raises the over-adjustment and collider-bias caveat discussed above. Fourth, very uncommon outcomes yielded wide CIs and unstable estimates; ECMO use within the schizophrenia/psychotic and PTSD groups involved too few events for stable or reportable estimation and is therefore not presented, and some estimates were non-estimable in the oldest age stratum. Fifth, race/ethnicity and hospital region were unavailable in the 2023 file and were handled via a 2016–22 sensitivity analysis. Sixth, we did not correct for multiple comparisons across the many outcome–exposure combinations, and secondary and rare-event associations should be regarded as hypothesis-generating; several very rare procedures were non-estimable and are reported as such. Seventh, restricting the cohort to Type 1 infarction excludes Type 2 events (I21.A); because several antipsychotic agents prolong the QT interval and carry arrhythmogenic potential in addition to their cardiometabolic effects,^32^ Type 2 infarction may be a mechanistically relevant presentation in this population that the present design does not capture. This analysis predates the Fifth Universal Definition of Myocardial Infarction,^45^ which replaces the numbered types with primary, secondary, and procedure-related categories; the cohort studied here corresponds to primary and the excluded group to secondary infarction. Eighth, the adjusted in-hospital mortality estimate for schizophrenia/psychotic disorders was sensitive to covariate specification, ranging from 0.86 in the expanded model to 1.20 in a parsimonious model, and no inference about in-hospital mortality should be drawn from it. Finally, all associations are observational and cannot establish causation.

## Conclusions

In this nationally representative analysis of AMI hospitalizations from 2016 through 2023, schizophrenia/psychotic disorders were consistently associated with markedly lower odds of coronary angiography, invasive management, and revascularization, with the largest deficit at first presentation. Associations with MCS were less consistent and did not reach significance after adjustment; the in-hospital mortality estimate was sensitive to model specification, and no inference about in-hospital mortality is drawn. These findings are observational and support further investigation of patient, clinician, and health-system factors contributing to differences in AMI management among patients with SMI.

## Supplementary Material

oeag171_Supplementary_Data

## Data Availability

The National Inpatient Sample is maintained by the Agency for Healthcare Research and Quality and is publicly available through the Healthcare Cost and Utilization Project (https://www.hcup-us.ahrq.gov/).

## References

[1] Martin SS, Aday AW, Allen NB, Almarzooq ZI, Anderson CAM, Arora P, Avery CL, Baker-Smith CM, Bansal N, Beaton AZ, Commodore-Mensah Y, Currie ME, Elkind MSV, Fan W, Generoso G, Gibbs BB, Heard DG, Hiremath S, Johansen MC, Kazi DS, Ko D, Leppert MH, Magnani JW, Michos ED, Mussolino ME, Parikh NI, Perman SM, Rezk-Hanna M, Roth GA, Shah NS, Springer MV, St-Onge MP, Thacker EL, Urbut SM, Van Spall HGC, Voeks JH, Whelton SP, Wong ND, Wong SS, Yaffe K, Palaniappan LP; American Heart Association Council on Epidemiology and Prevention Statistics Committee and Stroke Statistics Committee. 2025 Heart Disease and Stroke Statistics: a report of US and global data from the American Heart Association. Circulation 2025;151:e41–e660. 10.1161/CIR.0000000000001303 PMC12256702 39866113

[2] Levine GN, Cohen BE, Commodore-Mensah Y, Fleury J, Huffman JC, Khalid U, Labarthe DR, Lavretsky H, Michos ED, Spatz ES, Kubzansky LD. Psychological health, well-being, and the mind-heart-body connection: a scientific statement from the American Heart Association. Circulation 2021;143:e763–e783. 10.1161/CIR.0000000000000947 33486973

[3] Levine GN . The mind-heart-body connection. Circulation 2019;140:1363–1365. 10.1161/CIRCULATIONAHA.119.041914 31634016

[4] Lichtman JH, Froelicher ES, Blumenthal JA, Carney RM, Doering LV, Frasure-Smith N, Freedland KE, Jaffe AS, Leifheit-Limson EC, Sheps DS, Vaccarino V, Wulsin L; American Heart Association Statistics Committee of the Council on Epidemiology and Prevention and the Council on Cardiovascular and Stroke Nursing. Depression as a risk factor for poor prognosis among patients with acute coronary syndrome: systematic review and recommendations: a scientific statement from the American Heart Association. Circulation 2014;129:1350–1369. 10.1161/CIR.0000000000000019 24566200

[5] Goldstein BI, Carnethon MR, Matthews KA, McIntyre RS, Miller GE, Raghuveer G, Stoney CM, Wasiak H, McCrindle BW; American Heart Association Atherosclerosis; Hypertension and Obesity in Youth Committee of the Council on Cardiovascular Disease in the Young. Major depressive disorder and bipolar disorder predispose youth to accelerated atherosclerosis and early cardiovascular disease: a scientific statement from the American Heart Association. Circulation 2015;132:965–986. 10.1161/CIR.0000000000000229 26260736

[6] Gan Y, Gong Y, Tong X, Sun H, Cong Y, Dong X, Wang Y, Xu X, Yin X, Deng J, Li L, Cao S, Lu Z. Depression and the risk of coronary heart disease: a meta-analysis of prospective cohort studies. BMC Psychiatry 2014;14:371. 10.1186/s12888-014-0371-z PMC4336481 25540022

[7] Frasure-Smith N, Lespérance F. Depression and anxiety as predictors of 2-year cardiac events in patients with stable coronary artery disease. Arch Gen Psychiatry 2008;65:62–71. 10.1001/archgenpsychiatry.2007.4 18180430

[8] Wu S-I, Chen S-C, Liu S-I, Sun F-J, Juang JJM, Lee H-C, Kao K-L, Dewey ME, Prince M, Stewart R. Relative risk of acute myocardial infarction in people with schizophrenia and bipolar disorder: a population-based cohort study. PLoS One 2015;10:e0134763. 10.1371/journal.pone.0134763 PMC4536090 26270347

[9] Goldstein BI, Fagiolini A, Houck P, Kupfer DJ. Cardiovascular disease and hypertension among adults with bipolar I disorder in the United States. Bipolar Disord 2009;11:657–662. 10.1111/j.1399-5618.2009.00735.x PMC3401900 19689508

[10] Goldfarb M, De Hert M, Detraux J, Di Palo K, Munir H, Music S, Piña I, Ringen PA. Severe mental illness and cardiovascular disease: JACC state-of-the-art review. J Am Coll Cardiol 2022;80:918–933. 10.1016/j.jacc.2022.06.017 36007991

[11] Druss BG, Bradford DW, Rosenheck RA, Radford MJ, Krumholz HM. Mental disorders and use of cardiovascular procedures after myocardial infarction. JAMA 2000;283:506–511. 10.1001/jama.283.4.506 10659877

[12] Petersen LA, Normand SLT, Druss BG, Rosenheck RA. Process of care and outcome after acute myocardial infarction for patients with mental illness in the VA health care system: are there disparities? Health Serv Res 2003;38:41–63. 10.1111/1475-6773.00104 PMC1360873 12650380

[13] Laursen TM, Munk-Olsen T, Agerbo E, Gasse C, Mortensen PB. Somatic hospital contacts, invasive cardiac procedures, and mortality from heart disease in patients with severe mental disorder. Arch Gen Psychiatry 2009;66:713–720. 10.1001/archgenpsychiatry.2009.61 19581562

[14] Druss BG, Bradford WD, Rosenheck RA, Radford MJ, Krumholz HM. Quality of medical care and excess mortality in older patients with mental disorders. Arch Gen Psychiatry 2001;58:565–572. 10.1001/archpsyc.58.6.565 11386985

[15] Sreenivasan J, Khan MS, Khan SU, Hooda U, Aronow WS, Panza JA, Levine GN, Commodore-Mensah Y, Blumenthal RS, Michos ED. Mental health disorders among patients with acute myocardial infarction in the United States. Am J Prev Cardiol 2021;5:100133. 10.1016/j.ajpc.2020.100133 PMC8315415 34327485

[16] Mohamed MO, Rashid M, Farooq S, Siddiqui N, Parwani P, Shiers D, Thamman R, Gulati M, Shoaib A, Chew-Graham C, Mamas MA. Acute myocardial infarction in severe mental illness: prevalence, clinical outcomes, and process of care in U.S. hospitalizations. Can J Cardiol 2019;35:821–830. 10.1016/j.cjca.2019.04.021 31292080

[17] Fleetwood K, Wild SH, Smith DJ, Mercer SW, Licence K, Sudlow CLM, Jackson CA. Severe mental illness and mortality and coronary revascularisation following a myocardial infarction: a retrospective cohort study. BMC Med 2021;19:67. 10.1186/s12916-021-01937-2 PMC7983231 33745445

[18] Kugathasan P, Horsdal HT, Aagaard J, Jensen SE, Laursen TM, Nielsen RE. Association of secondary preventive cardiovascular treatment after myocardial infarction with mortality among patients with schizophrenia. JAMA Psychiatry 2018;75:1234–1240. 10.1001/jamapsychiatry.2018.2742 PMC6583028 30422158

[19] Khera R, Angraal S, Couch T, Welsh JW, Nallamothu BK, Girotra S, Chan PS, Krumholz HM. Adherence to methodological standards in research using the national inpatient sample. JAMA 2017;318:2011–2018. 10.1001/jama.2017.17653 PMC5742631 29183077

[20] Khera R, Krumholz HM. With great power comes great responsibility: big data research from the national inpatient sample. Circ Cardiovasc Qual Outcomes 2017;10:e003846. 10.1161/CIRCOUTCOMES.117.003846 PMC5728376 28705865

[21] Thygesen K, Alpert JS, Jaffe AS, Chaitman BR, Bax JJ, Morrow DA, White HD; ESC Scientific Document Group. Fourth universal definition of myocardial infarction (2018). Eur Heart J 2019;40:237–269. 10.1093/eurheartj/ehy462 30165617

[22] Elixhauser A, Steiner C, Harris DR, Coffey RM. Comorbidity measures for use with administrative data. Med Care 1998;36:8–27. 10.1097/00005650-199801000-00004 9431328

[23] Ruscio AM, Hallion LS, Lim CCW, Aguilar-Gaxiola S, Al-Hamzawi A, Alonso J, Andrade LH, Borges G, Bromet EJ, Bunting B, Caldas de Almeida JM, Demyttenaere K, Florescu S, de Girolamo G, Gureje O, Haro JM, He Y, Hinkov H, Hu C, de Jonge P, Karam EG, Lee S, Lepine J-P, Levinson D, Mneimneh Z, Navarro-Mateu F, Posada-Villa J, Slade T, Stein DJ, Torres Y, Uda H, Wojtyniak B, Kessler RC, Chatterji S, Scott KM. Cross-sectional comparison of the epidemiology of DSM-5 generalized anxiety disorder across the globe. JAMA Psychiatry 2017;74:465–475. 10.1001/jamapsychiatry.2017.0056 PMC5594751 28297020

[24] Brody DJ, Pratt LA, Hughes JP. Prevalence of Depression Among Adults Aged 20 and Over: United States, 2013–2016. NCHS Data Brief No. 303. Hyattsville, MD: National Center for Health Statistics; 2018. 29638213

[25] Brody DJ, Hughes JP. Depression Prevalence in Adolescents and Adults: United States, August 2021–August 2023. NCHS Data Brief No. 527. Hyattsville, MD: National Center for Health Statistics; 2025. 10.15620/cdc/174579 PMC12400127 40839857

[26] Davis KAS, Sudlow CLM, Hotopf M. Can mental health diagnoses in administrative data be used for research? A systematic review of the accuracy of routinely collected diagnoses. BMC Psychiatry 2016;16:263. 10.1186/s12888-016-0963-x PMC4960739 27455845

[27] Harpaz-Rotem I, Leslie DL, Martin A, Rosenheck RA. Changes in child and adolescent inpatient psychiatric admission diagnoses between 1995 and 2000. Soc Psychiatry Psychiatr Epidemiol 2005;40:642–647. 10.1007/s00127-005-0923-0 16133747

[28] Moreno C, Laje G, Blanco C, Jiang H, Schmidt AB, Olfson M. National trends in the outpatient diagnosis and treatment of bipolar disorder in youth. Arch Gen Psychiatry 2007;64:1032–1039. 10.1001/archpsyc.64.9.1032 17768268

[29] Schulman-Marcus J, Goyal P, Swaminathan RV, Feldman DN, Wong SC, Singh HS, Minutello RM, Bergman G, Kim LK. Comparison of trends in incidence, revascularization, and in-hospital mortality in ST-elevation myocardial infarction in patients with versus without severe mental illness. Am J Cardiol 2016;117:1405–1410. 10.1016/j.amjcard.2016.02.006 26956637

[30] Schulman-Marcus J, Shah T, Swaminathan RV, Feldman DN, Wong SC, Singh HS, Minutello RM, Bergman G, Kim LK. Comparison of recent trends in patients with and without major depression and acute ST-elevation myocardial infarction. Am J Cardiol 2016;118:779–784. 10.1016/j.amjcard.2016.06.051 27453511

[31] Solmi M, Fiedorowicz J, Poddighe L, Delogu M, Miola A, Høye A, Heiberg IH, Stubbs B, Smith L, Larsson H, Attar R, Nielsen RE, Cortese S, Shin JI, Fusar-Poli P, Firth J, Yatham LN, Carvalho AF, Castle DJ, Seeman MV, Correll CU. Disparities in screening and treatment of cardiovascular diseases in patients with mental disorders across the world: systematic review and meta-analysis of 47 observational studies. Am J Psychiatry 2021;178:793–803. 10.1176/appi.ajp.2021.21010031 34256605

[32] Ray WA, Chung CP, Murray KT, Hall K, Stein CM. Atypical antipsychotic drugs and the risk of sudden cardiac death. N Engl J Med 2009;360:225–235. 10.1056/NEJMoa0806994 PMC2713724 19144938

[33] Abrams TE, Vaughan-Sarrazin M, Rosenthal GE. Psychiatric comorbidity and mortality after acute myocardial infarction. Circ Cardiovasc Qual Outcomes 2009;2:213–220. 10.1161/CIRCOUTCOMES.108.829143 20031840

[34] Zen AL, Whooley MA, Zhao S, Cohen BE. Post-traumatic stress disorder is associated with poor health behaviors: findings from the heart and soul study. Health Psychol 2012;31:194–201. 10.1037/a0025989 PMC3295904 22023435

[35] Shah AJ, Lampert R, Goldberg J, Veledar E, Bremner JD, Vaccarino V. Posttraumatic stress disorder and impaired autonomic modulation in male twins. Biol Psychiatry 2013;73:1103–1110. 10.1016/j.biopsych.2013.01.019 PMC3648627 23434412

[36] Passos IC, Vasconcelos-Moreno MP, Costa LG, Kunz M, Brietzke E, Quevedo J, Salum G, Magalhães PV, Kapczinski F, Kauer-Sant'Anna M. Inflammatory markers in post-traumatic stress disorder: a systematic review, meta-analysis, and meta-regression. Lancet Psychiatry 2015;2:1002–1012. 10.1016/S2215-0366(15)00309-0 26544749

[37] Lima BB, Hammadah M, Pearce BD, Shah A, Moazzami K, Kim JH, Sullivan S, Levantsevych O, Lewis TT, Weng L, Elon L, Li L, Raggi P, Bremner JD, Quyyumi A, Vaccarino V. Association of posttraumatic stress disorder with mental stress–induced myocardial ischemia in adults after myocardial infarction. JAMA Netw Open 2020;3:e202734. 10.1001/jamanetworkopen.2020.2734 PMC7156990 32286655

[38] Sreenivasan J, Kaul R, Khan MS, Malik A, Usman MS, Michos ED. Mental health disorders and readmissions following acute myocardial infarction in the United States. Sci Rep 2022;12:3327. 10.1038/s41598-022-07234-z PMC8885687 35228619

[39] Ahmedani BK, Solberg LI, Copeland LA, Fang-Hollingsworth Y, Stewart C, Hu J, Nerenz DR, Williams LK, Cassidy-Bushrow AE, Waxmonsky J, Lu CY, Waitzfelder BE, Owen-Smith AA, Coleman KJ, Lynch FL, Ahmed AT, Beck A, Rossom RC, Simon GE. Psychiatric comorbidity and 30-day readmissions after hospitalization for heart failure, AMI, and pneumonia. Psychiatr Serv 2015;66:134–140. 10.1176/appi.ps.201300518 PMC4315504 25642610

[40] Chan JKN, Chu RST, Hung C, Law JWY, Wong CSM, Chang WC. Mortality, revascularization, and cardioprotective pharmacotherapy after acute coronary syndrome in patients with severe mental illness: a systematic review and meta-analysis. Schizophr Bull 2022;48:981–998. 10.1093/schbul/sbac070 PMC9434477 35786737

[41] Van Doornum S, Bohensky M, Tacey MA, Brand CA, Sundararajan V, Wicks IP. Increased 30-day and 1-year mortality rates and lower coronary revascularisation rates following acute myocardial infarction in patients with autoimmune rheumatic disease. Arthritis Res Ther 2015;17:38. 10.1186/s13075-015-0552-2 PMC4372281 25879786

[42] Bueno H, Deaton C, Farrero M, Forsyth Faye, Braunschweig F, Buccheri S, Dragan S, Gevaert S, Held C, Kurpas D, Ladwig K-H, Lionis CD, Maas AHEM, Merșa CO, Mindham R, Pedersen SS, Rojnic Kuzman M, Szmit S, Taylor RS, Uchmanowicz I, Vilchinsky N; ESC Scientific Document Group. 2025 ESC Clinical Consensus Statement on mental health and cardiovascular disease: developed under the auspices of the ESC Clinical Practice Guidelines Committee. Eur Heart J 2025;46:4156–4225. 10.1093/eurheartj/ehaf191 40878270

[43] Lavie CJ, Menezes AR, De Schutter A, Milani RV, Blumenthal JA. Impact of cardiac rehabilitation and exercise training on psychological risk factors and subsequent prognosis in patients with cardiovascular disease. Can J Cardiol 2016;32:S365–S373. 10.1016/j.cjca.2016.07.508 27692117

[44] Mitchell AJ, Lord O, Malone D. Differences in the prescribing of medication for physical disorders in individuals with v. without mental illness: meta-analysis. Br J Psychiatry 2012;201:435–443. 10.1192/bjp.bp.111.094532 23209089

[45] Mills NL, Newby LK, Zaman S, Almahmeed WA, Bucciarelli-Ducci C, Colvin MM, Dangas GD, DeVon HA, Dharmakumar R, Gaziano TA, Hausenloy DJ, Jeilan M, Koskinas KC, Lindahl B, Motovska Z, Rubini Gimenez M, Salvati AM, Tamis-Holland JE; ESC/ACC/AHA/WHF Universal Definition Document Advisory Group. Fifth universal definition of myocardial infarction (2026). Eur Heart J 2026;28:ehag101. 10.1093/eurheartj/ehag101 42661425

