# Supplementary material for: Psychiatric comorbidity and disparities in the invasive management of acute myocardial infarction: a national analysis, 2016–23
Source: Eur Heart J Open. 2026 Oct 13;6(6):oeag171. doi: 10.1093/ehjopen/oeag171 (PMC13759986; doi:10.1093/ehjopen/oeag171)
Supplement: Supplementary file 1 [file oeag171_supplementary_data.docx]

# Supplementary Appendix

*Reference categories are hospitalizations without the respective disorder unless noted. In outcome tables, each cell is formatted as adjusted OR (95% CI): P value; cells whose 95% CI includes 1.0 (not statistically significant) are shaded gray and italicized.*

**eTable 1. ICD-10-CM (diagnosis) and ICD-10-PCS (procedure) codebook.**

| **Variable** | **Codes** |
| --- | --- |
| **Exposure — mental health disorders (secondary diagnosis fields)** | |
| Major depression | F32, F33 |
| Anxiety disorders | F41 |
| Bipolar disorder | F31 |
| Schizophrenia/other psychotic disorders | F20–F29 |
| Post-traumatic stress disorder | F43.1 |
| **Cohort — acute myocardial infarction (principal diagnosis)** | |
| AMI (inclusion) | I21.x, I22.x |
| Type 2 MI (exclusion) | I21.A |
| STEMI | I21.0–I21.3, I22.0, I22.1, I22.8, I22.9 |
| NSTEMI | I21.4 |
| **Comorbidities (any diagnosis field; Elixhauser/AHRQ-aligned)** | |
| Obesity | E66 |
| Anemia | D50–D53 |
| Diabetes mellitus | E08–E13 |
| Hypertension | I10–I16 |
| Hyperlipidemia | E78.0, E78.1, E78.2, E78.4, E78.5 |
| Chronic liver disease | K70, K72, K74, B18, I85 |
| Chronic kidney disease | N18, N19 |
| Dialysis dependence | Z99.2 |
| Pulmonary vascular disease | I27, I28 |
| Chronic obstructive pulmonary disease | J44 |
| Peripheral arterial disease | I70, I71, I73, I77 |
| Dementia | G30, F01, F02, F03 |
| Prior myocardial infarction | I25.2 |
| Prior PCI | Z95.5 |
| Prior CABG | Z95.1 |
| Prior stroke | I69.4, Z86.73 |
| Atrial fibrillation/flutter | I48 |
| Alcohol use disorder | F10 |
| Drug use disorder | F11, F12, F13, F14, F15, F16, F18, F19 (tobacco F17 excluded) |
| **Coronary procedures (ICD-10-PCS)** | |
| Coronary angiography | B21 |
| PCI | 027 (dilation of the coronary artery) with device value 3 or 4 |
| CABG | 0210, 0211, 0212, 0213 |
| Coronary thrombectomy | 02C0–02C3 |
| **Mechanical circulatory support and cardiac procedures (ICD-10-PCS)** | |
| Intra-aortic balloon pump (IABP) | 5A02210, 5A02110 |
| Percutaneous LVAD (pLVAD) | 5A0221D, 5A0211D, 5A02216, 5A02116, 02HA0RJ, 02HA3RJ, 02HA4RJ |
| Extracorporeal membrane oxygenation (ECMO) | 5A15223, 5A1522F, 5A1522G, 5A1522H |
| Durable LVAD | 02HA0QZ, 02HA3QZ, 02HA4QZ |
| Heart transplant | 02YA0Z0, 02YA0Z1, 02YA0Z2 |
| Right heart catheterization | 4A023N6, 4A023N8 |
| Left heart catheterization | 4A023N7, 4A023N8 |
| **Organ support (ICD-10-PCS)** | |
| Invasive mechanical ventilation | 5A1935Z, 5A1945Z, 5A1955Z |
| Acute hemodialysis | 5A1D00Z, 5A1D60Z, 5A1D70Z, 5A1D80Z, 5A1D90Z |
| **Complications and severity (diagnosis fields)** | |
| Cardiogenic shock | R57.0 |
| Cardiac arrest | I46.2, I46.8, I46.9 |
| Ischemic stroke | I63 |
| Intracranial hemorrhage | I61, I62 |
| Acute kidney injury | N17 |
| Major bleeding | K66.1, K92.2, R58 |
| Post-MI ventricular septal defect | I23.2 |
| Papillary muscle rupture | I23.5 |
| Hemopericardium | I23.0 |
| **Care processes and pandemic covariate** | |
| Palliative-care encounter | Z51.5 |
| Do-not-resuscitate status | Z66 |
| COVID-19 (COVID-era models; from 1 April 2020) | U07.1 |

*Diagnoses use ICD-10-CM; procedures use ICD-10-PCS. The AMI cohort was defined on the principal diagnosis; mental health disorders and comorbidities were ascertained from secondary/any diagnosis fields. Derived variables: any revascularization = PCI or CABG; serious mental illness = schizophrenia/psychotic or bipolar disorder; invasive management and any MCS are composites of the procedures listed above. ECMO and hemodialysis lists include both legacy and current ICD-10-PCS codes because NIS files are calendar years that include the 1 October ICD-10 update. Anxiety disorders were defined by F41 (including generalized anxiety and panic disorders); phobic (F40) and acute stress (F43.0) disorders were not included, reflecting a focus on the predominant anxiety-disorder category. Code 4A023N8 denotes combined right- and left-heart catheterization and therefore appears under both catheterization variables. Transcribed from the analytic extraction script (NIS 2016–2023).*

**eTable 2. Secular coding comparator conditions: prevalence trends, 2016–2023.**

| **Variable** | **2016** | **2017** | **2018** | **2019** | **2020** | **2021** | **2022** | **2023** | **APC** | **95% CI** | **P** |
| --- | --- | --- | --- | --- | --- | --- | --- | --- | --- | --- | --- |
| Hyperlipidemia | 66.1 | 66.5 | 67.6 | 69.1 | 70.6 | 70.9 | 71.7 | 72.5 | 4.7 | 4.3, 5.2 | <0.001 |
| Anemia | 3.8 | 3.7 | 3.5 | 3.6 | 3.7 | 3.9 | 4.1 | 4.3 | 2.2 | 1.6, 2.8 | <0.001 |
| Peripheral arterial disease | 12.1 | 10.8 | 9.5 | 9.5 | 9.5 | 9.7 | 9.8 | 9.9 | -2.6 | -3.1, -2.2 | <0.001 |

*APC = annual percent change in prevalence odds. These comparator conditions index background coding drift for comparison with the MHD trends in eTable 3; they are not formal causal-inference controls.*

**eTable 3. Year-by-year prevalence (%) of each mental health disorder among AMI hospitalizations, 2016–2023.**

| **Variable** | **2016** | **2017** | **2018** | **2019** | **2020** | **2021** | **2022** | **2023** | **APC** | **95% CI** | **P** |
| --- | --- | --- | --- | --- | --- | --- | --- | --- | --- | --- | --- |
| Major depression | 8.5 | 8.9 | 9.0 | 9.2 | 9.5 | 9.8 | 9.6 | 10.1 | 2.5 | 2.0, 3.0 | <0.001 |
| Anxiety disorders | 10.1 | 10.7 | 10.8 | 11.2 | 11.9 | 12.5 | 12.3 | 12.7 | 3.8 | 3.4, 4.2 | <0.001 |
| Bipolar disorder | 1.1 | 1.2 | 1.2 | 1.2 | 1.4 | 1.3 | 1.3 | 1.4 | 3.6 | 2.8, 4.5 | <0.001 |
| Schizophrenia/psychotic | 0.7 | 0.8 | 0.7 | 0.7 | 0.8 | 0.8 | 0.8 | 0.7 | 0.6 | -0.5, 1.7 | 0.316 |
| PTSD | 0.5 | 0.6 | 0.7 | 0.7 | 0.8 | 0.8 | 0.9 | 1.1 | 10.3 | 9.0, 11.6 | <0.001 |

*Weighted prevalence per year with APC (annual percent change in prevalence odds) and P for trend.*

**eTable 4. Full adjusted outcome model — all mental health disorders across all in-hospital outcomes (main model, 2016–2023).**

| **Outcome** | **Major depression** | **Anxiety disorders** | **Bipolar disorder** | **Schizophrenia/psychotic** | **PTSD** |
| --- | --- | --- | --- | --- | --- |
| Any revascularization | 0.94 (0.92-0.95)*: P<.001* | *0.98 (0.97-1.00): P=.06* | 0.81 (0.78-0.85)*: P<.001* | 0.56 (0.53-0.60)*: P<.001* | 0.93 (0.89-0.98)*: P=.009* |
| PCI | 0.97 (0.95-0.98)*: P<.001* | 0.92 (0.90-0.93)*: P<.001* | 0.85 (0.82-0.89)*: P<.001* | 0.61 (0.58-0.65)*: P<.001* | 0.93 (0.88-0.98)*: P=.006* |
| CABG | 0.93 (0.91-0.96)*: P<.001* | 1.22 (1.19-1.25)*: P<.001* | 0.88 (0.82-0.94)*: P<.001* | 0.76 (0.69-0.84)*: P<.001* | *1.00 (0.93-1.09): P=.94* |
| Invasive management | 0.98 (0.96-0.99)*: P=.01* | *1.02 (1.00-1.04): P=.05* | 0.85 (0.81-0.89)*: P<.001* | 0.52 (0.49-0.55)*: P<.001* | *1.00 (0.95-1.07): P=.89* |
| Coronary angiography | *0.99 (0.97-1.01): P=.42* | 1.03 (1.01-1.05)*: P=.002* | 0.86 (0.83-0.90)*: P<.001* | 0.55 (0.53-0.58)*: P<.001* | *1.01 (0.95-1.06): P=.85* |
| Left heart catheterization | 0.96 (0.94-0.98)*: P<.001* | *1.02 (1.00-1.03): P=.03* | 0.88 (0.84-0.92)*: P<.001* | 0.60 (0.57-0.63)*: P<.001* | 0.93 (0.88-0.98)*: P=.007* |
| Right heart catheterization | 0.95 (0.92-0.99)*: P=.02* | 0.96 (0.92-0.99)*: P=.03* | *0.94 (0.85-1.04): P=.26* | 0.84 (0.74-0.96)*: P=.010* | *0.94 (0.82-1.07): P=.33* |
| Coronary thrombectomy | 0.94 (0.91-0.98)*: P=.002* | *0.98 (0.94-1.01): P=.16* | 0.90 (0.82-0.98)*: P=.02* | 0.75 (0.66-0.85)*: P<.001* | *0.89 (0.80-1.00): P=.05* |
| Intravascular ultrasound | *1.04 (1.00-1.07): P=.02* | *1.00 (0.97-1.03): P=.82* | 0.91 (0.84-0.99)*: P=.03* | 0.76 (0.68-0.86)*: P<.001* | 1.27 (1.16-1.38)*: P<.001* |
| Fibrinolysis | *0.90 (0.81-1.00): P=.05* | *1.05 (0.96-1.16): P=.28* | *0.94 (0.73-1.20): P=.60* | *0.95 (0.69-1.31): P=.76* | *0.97 (0.70-1.33): P=.83* |
| In-hospital mortality | 0.86 (0.82-0.90)*: P<.001* | 0.81 (0.78-0.84)*: P<.001* | 0.81 (0.72-0.91)*: P<.001* | *1.11 (0.98-1.26): P=.09* | 0.69 (0.58-0.81)*: P<.001* |
| Any MCS | 0.91 (0.88-0.94)*: P<.001* | *1.02 (0.99-1.05): P=.26* | 0.78 (0.71-0.86)*: P<.001* | *0.89 (0.79-1.00): P=.05* | 0.83 (0.73-0.94)*: P=.002* |
| IABP | 0.94 (0.90-0.97)*: P=.002* | 1.05 (1.01-1.09)*: P=.02* | 0.80 (0.72-0.90)*: P<.001* | *0.94 (0.83-1.08): P=.41* | *0.90 (0.79-1.02): P=.11* |
| Percutaneous LVAD | 0.86 (0.80-0.92)*: P<.001* | *0.95 (0.89-1.01): P=.09* | 0.76 (0.64-0.92)*: P=.003* | *0.81 (0.65-1.01): P=.07* | 0.61 (0.47-0.79)*: P<.001* |
| ECMO | *0.84 (0.70-1.01): P=.06* | 0.81 (0.68-0.95)*: P=.01* | *0.66 (0.41-1.08): P=.10* | NR | *NR* |
| Durable LVAD | 1.78 (1.21-2.62)*: P=.003* | 0.60 (0.38-0.96)*: P=.03* | NE | *1.05 (0.28-3.95): P=.94* | NE |
| Heart transplant | NE | *0.99 (0.32-3.06): P=.99* | NE | NE | NE |
| Invasive mechanical ventilation | 0.87 (0.84-0.91)*: P<.001* | 0.89 (0.86-0.92)*: P<.001* | 0.83 (0.76-0.91)*: P<.001* | 1.37 (1.24-1.51)*: P<.001* | 0.84 (0.74-0.95)*: P=.004* |
| Noninvasive ventilation | 1.08 (1.03-1.12)*: P<.001* | 1.22 (1.17-1.27)*: P<.001* | *1.06 (0.96-1.18): P=.27* | 1.24 (1.10-1.40)*: P<.001* | *0.93 (0.80-1.07): P=.30* |
| Acute hemodialysis | *0.97 (0.92-1.02): P=.29* | 0.94 (0.89-0.99)*: P=.02* | 0.76 (0.66-0.88)*: P<.001* | *0.94 (0.80-1.11): P=.47* | 0.69 (0.56-0.85)*: P<.001* |
| Blood transfusion | *0.97 (0.94-1.01): P=.19* | *1.03 (0.99-1.07): P=.08* | 0.87 (0.78-0.98)*: P=.02* | 1.15 (1.01-1.30)*: P=.03* | 0.77 (0.66-0.90)*: P<.001* |
| Any complication | 0.96 (0.94-0.98)*: P<.001* | 0.94 (0.92-0.95)*: P<.001* | *0.97 (0.93-1.02): P=.29* | 1.25 (1.18-1.32)*: P<.001* | 0.92 (0.86-0.98)*: P=.01* |
| Cardiogenic shock | 0.90 (0.87-0.94)*: P<.001* | *0.97 (0.94-1.00): P=.05* | 0.85 (0.78-0.93)*: P<.001* | *1.03 (0.93-1.15): P=.54* | 0.83 (0.73-0.93)*: P=.002* |
| Cardiac arrest | 0.84 (0.80-0.88)*: P<.001* | 0.87 (0.83-0.90)*: P<.001* | 0.77 (0.69-0.87)*: P<.001* | 1.16 (1.02-1.32)*: P=.02* | *0.99 (0.86-1.15): P=.94* |
| Acute kidney injury | 0.97 (0.95-0.99)*: P=.004* | 0.95 (0.93-0.96)*: P<.001* | *1.00 (0.95-1.06): P=.86* | 1.27 (1.19-1.35)*: P<.001* | 0.92 (0.86-0.98)*: P=.02* |
| Major bleeding | *0.96 (0.88-1.04): P=.31* | 0.85 (0.78-0.92)*: P<.001* | *1.02 (0.82-1.25): P=.88* | 1.39 (1.11-1.73)*: P=.004* | 0.60 (0.42-0.85)*: P=.005* |
| Ischemic stroke | 1.12 (1.05-1.19)*: P<.001* | 0.78 (0.73-0.83)*: P<.001* | 0.82 (0.68-0.99)*: P=.04* | *1.11 (0.91-1.36): P=.29* | 0.69 (0.52-0.89)*: P=.005* |
| Intracranial hemorrhage | *1.03 (0.86-1.24): P=.74* | 0.69 (0.57-0.84)*: P<.001* | *1.01 (0.64-1.60): P=.96* | *1.01 (0.59-1.72): P=.97* | *0.73 (0.36-1.50): P=.39* |
| Acute respiratory failure | *1.00 (0.97-1.02): P=.82* | 1.08 (1.06-1.11)*: P<.001* | *0.94 (0.89-1.00): P=.05* | 1.18 (1.10-1.27)*: P<.001* | 0.85 (0.79-0.92)*: P<.001* |
| Acute hepatic failure | 0.71 (0.64-0.78)*: P<.001* | 0.74 (0.67-0.81)*: P<.001* | 0.54 (0.43-0.68)*: P<.001* | 0.69 (0.52-0.90)*: P=.007* | 0.54 (0.39-0.73)*: P<.001* |
| Hematologic failure/DIC | *0.99 (0.95-1.02): P=.51* | 1.06 (1.03-1.10)*: P<.001* | *0.95 (0.86-1.05): P=.32* | *1.11 (0.99-1.24): P=.08* | 0.88 (0.77-0.99)*: P=.04* |
| Acute neurologic failure | 1.13 (1.09-1.18)*: P<.001* | *0.98 (0.94-1.01): P=.22* | 1.18 (1.07-1.29)*: P<.001* | 2.08 (1.91-2.28)*: P<.001* | *0.93 (0.81-1.06): P=.27* |
| Palliative-care encounter | *1.03 (0.99-1.08): P=.12* | 1.13 (1.09-1.18)*: P<.001* | *1.00 (0.88-1.14): P=.97* | 1.27 (1.12-1.45)*: P<.001* | 0.82 (0.68-0.98)*: P=.03* |
| DNR status | 1.27 (1.23-1.30)*: P<.001* | 1.07 (1.04-1.10)*: P<.001* | 1.32 (1.21-1.44)*: P<.001* | 1.35 (1.22-1.49)*: P<.001* | *1.01 (0.90-1.14): P=.85* |
| Access-site complication | *1.07 (0.93-1.24): P=.34* | *0.95 (0.83-1.10): P=.51* | *1.05 (0.72-1.54): P=.80* | *1.50 (0.99-2.29): P=.06* | *0.97 (0.58-1.61): P=.90* |
| Acute limb ischemia | *0.95 (0.84-1.07): P=.37* | *0.99 (0.89-1.11): P=.85* | *0.79 (0.57-1.10): P=.16* | *0.91 (0.61-1.36): P=.64* | *0.75 (0.48-1.17): P=.21* |
| Hemolysis | *0.73 (0.35-1.50): P=.39* | *1.34 (0.77-2.33): P=.29* | NE | NE | NE |

*Adjusted OR (95% CI): P value for every outcome; reference = hospitalizations without the respective disorder. NR, not reportable: the underlying cell contains 10 or fewer unweighted discharges and is therefore suppressed in accordance with the HCUP Data Use Agreement (see eTable 17).*

**eTable 5a. Temporal trend in any revascularization by the MHD group, 2016–2023.**

| **Group** | **2016** | **2017** | **2018** | **2019** | **2020** | **2021** | **2022** | **2023** | **APC** | **95% CI** | **P** | **P-int** |
| --- | --- | --- | --- | --- | --- | --- | --- | --- | --- | --- | --- | --- |
| Overall | 55.8 | 56.1 | 59.1 | 59.9 | 60.7 | 59.5 | 58.5 | 59.4 | 2.0 | 1.6, 2.5 | <0.001 |  |
| No MHD | 57.0 | 57.3 | 60.1 | 60.9 | 61.9 | 60.7 | 59.6 | 60.4 | 2.0 | 1.5, 2.4 | <0.001 |  |
| Major depression | 48.3 | 49.0 | 53.8 | 54.2 | 55.6 | 53.9 | 53.0 | 54.7 | 3.2 | 2.5, 4.0 | <0.001 | <0.001 |
| Bipolar disorder | 49.9 | 49.2 | 52.7 | 52.5 | 52.0 | 50.7 | 48.9 | 50.9 | -0.1 | -1.6, 1.5 | 0.932 | 0.008 |
| Anxiety disorders | 51.0 | 51.0 | 55.8 | 56.1 | 56.6 | 55.6 | 54.5 | 56.6 | 2.7 | 2.0, 3.4 | <0.001 | 0.021 |
| Schizophrenia/psychotic | 39.7 | 36.2 | 38.2 | 43.0 | 40.2 | 40.0 | 41.6 | 41.1 | 2.0 | -0.2, 4.3 | 0.071 | 0.992 |
| PTSD | 57.6 | 54.1 | 60.2 | 62.2 | 56.4 | 59.8 | 54.0 | 55.3 | -1.6 | -3.6, 0.5 | 0.145 | <0.001 |

*Rates (%) by year; APC and MHD×year interaction (P-int).*

**eTable 5b. Temporal trend in PCI by MHD group, 2016–2023.**

| **Group** | **2016** | **2017** | **2018** | **2019** | **2020** | **2021** | **2022** | **2023** | **APC** | **95% CI** | **P** | **P-int** |
| --- | --- | --- | --- | --- | --- | --- | --- | --- | --- | --- | --- | --- |
| Overall | 47.7 | 47.9 | 50.5 | 51.2 | 52.1 | 50.9 | 49.8 | 51.1 | 1.7 | 1.4, 2.1 | <0.001 |  |
| No MHD | 48.9 | 49.2 | 51.7 | 52.4 | 53.4 | 52.2 | 51.1 | 52.2 | 1.8 | 1.4, 2.1 | <0.001 |  |
| Major depression | 40.7 | 41.2 | 45.5 | 45.3 | 47.1 | 45.3 | 44.3 | 46.1 | 2.7 | 2.0, 3.4 | <0.001 | 0.002 |
| Bipolar disorder | 42.0 | 41.7 | 44.9 | 44.6 | 44.9 | 42.8 | 41.2 | 43.4 | 0.1 | -1.5, 1.7 | 0.936 | 0.035 |
| Anxiety disorders | 42.4 | 42.1 | 46.5 | 46.0 | 47.0 | 45.5 | 44.8 | 47.0 | 2.1 | 1.5, 2.7 | <0.001 | 0.267 |
| Schizophrenia/psychotic | 32.4 | 31.3 | 31.3 | 35.3 | 35.3 | 33.3 | 34.9 | 35.4 | 2.5 | 0.2, 4.8 | 0.032 | 0.519 |
| PTSD | 47.3 | 44.0 | 48.3 | 52.3 | 48.0 | 49.3 | 45.4 | 45.6 | -0.7 | -2.8, 1.3 | 0.481 | 0.017 |

*Rates (%) by year; APC and MHD×year interaction (P-int).*

**eTable 5c. Temporal trend in CABG by MHD group, 2016–2023.**

| **Group** | **2016** | **2017** | **2018** | **2019** | **2020** | **2021** | **2022** | **2023** | **APC** | **95% CI** | **P** | **P-int** |
| --- | --- | --- | --- | --- | --- | --- | --- | --- | --- | --- | --- | --- |
| Overall | 8.8 | 8.8 | 9.2 | 9.4 | 9.2 | 9.2 | 9.1 | 8.8 | 0.4 | -0.4, 1.1 | 0.337 |  |
| No MHD | 8.8 | 8.7 | 9.1 | 9.2 | 9.1 | 9.0 | 9.0 | 8.6 | 0.1 | -0.6, 0.9 | 0.748 |  |
| Major depression | 8.3 | 8.3 | 8.9 | 9.3 | 9.1 | 9.0 | 9.0 | 9.0 | 1.4 | 0.1, 2.6 | 0.028 | 0.048 |
| Bipolar disorder | 8.5 | 8.4 | 8.0 | 8.6 | 7.6 | 8.2 | 8.1 | 7.7 | -1.1 | -4.0, 1.8 | 0.447 | 0.297 |
| Anxiety disorders | 9.4 | 9.5 | 9.9 | 10.7 | 10.2 | 10.6 | 10.2 | 10.2 | 1.4 | 0.3, 2.5 | 0.012 | 0.011 |
| Schizophrenia/psychotic | 7.9 | 5.2 | 7.3 | 8.7 | 5.2 | 7.1 | 7.2 | 5.7 | -1.6 | -5.5, 2.4 | 0.418 | 0.317 |
| PTSD | 11.2 | 10.5 | 12.6 | 10.7 | 8.9 | 11.1 | 9.0 | 10.0 | -2.8 | -6.0, 0.5 | 0.092 | 0.054 |

*Rates (%) by year; APC and MHD×year interaction (P-int).*

**eTable 5d. Temporal trend in in-hospital mortality by MHD group, 2016–2023.**

| **Group** | **2016** | **2017** | **2018** | **2019** | **2020** | **2021** | **2022** | **2023** | **APC** | **95% CI** | **P** | **P-int** |
| --- | --- | --- | --- | --- | --- | --- | --- | --- | --- | --- | --- | --- |
| Overall | 4.7 | 4.7 | 4.7 | 4.6 | 5.0 | 5.0 | 4.8 | 4.6 | 0.2 | -0.2, 0.7 | 0.264 |  |
| No MHD | 5.0 | 5.0 | 4.9 | 4.8 | 5.3 | 5.4 | 5.2 | 4.8 | 0.5 | -0.0, 0.9 | 0.055 |  |
| Major depression | 3.7 | 3.7 | 3.5 | 3.7 | 3.6 | 3.4 | 3.5 | 3.5 | -0.8 | -2.3, 0.8 | 0.337 | 0.164 |
| Bipolar disorder | 3.7 | 2.2 | 2.3 | 3.4 | 2.8 | 2.7 | 3.1 | 2.6 | -1.3 | -5.8, 3.4 | 0.585 | 0.508 |
| Anxiety disorders | 3.0 | 3.2 | 3.2 | 3.1 | 3.6 | 3.2 | 3.1 | 2.9 | -0.5 | -1.9, 1.0 | 0.504 | 0.212 |
| Schizophrenia/psychotic | 3.8 | 4.4 | 4.4 | 6.5 | 5.8 | 3.7 | 4.5 | 5.2 | 2.1 | -2.5, 6.9 | 0.381 | 0.44 |
| PTSD | 1.7 | 2.1 | 1.3 | 2.6 | 2.5 | 2.7 | 2.1 | 2.7 | 5.9 | -0.9, 13.3 | 0.091 | 0.108 |

*Rates (%) by year; APC and MHD×year interaction (P-int).*

**eTable 5e. Temporal trend in any MCS by MHD group, 2016–2023.**

| **Group** | **2016** | **2017** | **2018** | **2019** | **2020** | **2021** | **2022** | **2023** | **APC** | **95% CI** | **P** | **P-int** |
| --- | --- | --- | --- | --- | --- | --- | --- | --- | --- | --- | --- | --- |
| Overall | 4.9 | 5.1 | 5.6 | 5.7 | 5.9 | 5.9 | 5.8 | 5.9 | 2.6 | 2.0, 3.2 | <0.001 |  |
| No MHD | 5.0 | 5.3 | 5.8 | 6.0 | 6.2 | 6.1 | 6.0 | 6.1 | 2.6 | 2.0, 3.3 | <0.001 |  |
| Major depression | 3.8 | 3.8 | 4.5 | 4.5 | 4.5 | 4.5 | 4.9 | 4.9 | 4.0 | 2.4, 5.5 | <0.001 | 0.071 |
| Bipolar disorder | 3.6 | 4.0 | 3.9 | 4.0 | 3.7 | 3.9 | 4.3 | 3.7 | 0.6 | -3.4, 4.7 | 0.768 | 0.319 |
| Anxiety disorders | 4.1 | 4.2 | 4.6 | 4.8 | 4.7 | 4.9 | 5.0 | 4.6 | 2.4 | 1.0, 3.7 | <0.001 | 0.565 |
| Schizophrenia/psychotic | 4.3 | 4.9 | 3.5 | 4.8 | 5.3 | 5.7 | 4.4 | 3.1 | -1.1 | -5.6, 3.7 | 0.653 | 0.123 |
| PTSD | 4.6 | 3.5 | 4.3 | 4.1 | 4.4 | 5.5 | 4.2 | 3.4 | -0.7 | -5.5, 4.4 | 0.789 | 0.192 |

*Rates (%) by year; APC and MHD×year interaction (P-int).*

**eTable 5f. Temporal trend in IABP by MHD group, 2016–2023.**

| **Group** | **2016** | **2017** | **2018** | **2019** | **2020** | **2021** | **2022** | **2023** | **APC** | **95% CI** | **P** | **P-int** |
| --- | --- | --- | --- | --- | --- | --- | --- | --- | --- | --- | --- | --- |
| Overall | 4.1 | 4.0 | 4.1 | 4.1 | 4.2 | 4.1 | 3.9 | 3.7 | -0.8 | -1.5, -0.1 | 0.028 |  |
| No MHD | 4.2 | 4.1 | 4.2 | 4.2 | 4.4 | 4.3 | 4.0 | 3.8 | -0.9 | -1.6, -0.2 | 0.016 |  |
| Major depression | 3.2 | 3.1 | 3.3 | 3.3 | 3.3 | 3.4 | 3.5 | 3.3 | 1.2 | -0.5, 2.9 | 0.182 | 0.016 |
| Bipolar disorder | 3.0 | 3.2 | 2.9 | 3.1 | 3.2 | 2.4 | 2.7 | 2.6 | -3.1 | -7.4, 1.5 | 0.181 | 0.306 |
| Anxiety disorders | 3.6 | 3.4 | 3.5 | 3.5 | 3.5 | 3.6 | 3.6 | 3.2 | -0.6 | -2.2, 0.9 | 0.418 | 0.913 |
| Schizophrenia/psychotic | 3.8 | 4.0 | 3.0 | 3.9 | 3.3 | 4.6 | 3.0 | 1.5 | -5.7 | -10.7, -0.5 | 0.031 | 0.06 |
| PTSD | 4.1 | 3.1 | 3.6 | 3.2 | 3.5 | 4.1 | 3.2 | 2.4 | -3.8 | -8.9, 1.6 | 0.16 | 0.254 |

*Rates (%) by year; APC and MHD×year interaction (P-int).*

**eTable 5g. Temporal trend in percutaneous LVAD by MHD group, 2016–2023.**

| **Group** | **2016** | **2017** | **2018** | **2019** | **2020** | **2021** | **2022** | **2023** | **APC** | **95% CI** | **P** | **P-int** |
| --- | --- | --- | --- | --- | --- | --- | --- | --- | --- | --- | --- | --- |
| Overall | 0.9 | 1.2 | 1.7 | 1.8 | 1.8 | 1.9 | 2.0 | 2.2 | 11.2 | 10.1, 12.3 | <0.001 |  |
| No MHD | 0.9 | 1.2 | 1.8 | 1.9 | 1.9 | 2.0 | 2.1 | 2.4 | 11.2 | 10.1, 12.4 | <0.001 |  |
| Major depression | 0.6 | 0.8 | 1.3 | 1.3 | 1.3 | 1.3 | 1.4 | 1.7 | 11.7 | 8.7, 14.7 | <0.001 | 0.773 |
| Bipolar disorder | NR | 0.9 | 0.9 | 1.0 | NR | 1.4 | 1.6 | 1.4 | 11.9 | 3.7, 20.8 | 0.004 | 0.871 |
| Anxiety disorders | 0.5 | 0.8 | 1.3 | 1.3 | 1.3 | 1.4 | 1.6 | 1.6 | 12.2 | 9.5, 15.0 | <0.001 | 0.459 |
| Schizophrenia/psychotic | NR | 1.1 | NR | 1.3 | 2.0 | 1.5 | 1.5 | 1.5 | 12.8 | 3.3, 23.2 | 0.007 | 0.749 |
| PTSD | NR | NR | NR | NR | NR | 1.5 | 1.1 | 0.9 | 11.3 | 0.1, 23.8 | 0.049 | 0.992 |

*Rates (%) by year; APC and MHD×year interaction (P-int). Annual cells are suppressed individually wherever the underlying count is at or below the threshold; where a single suppression within a row would leave the value recoverable from the group total in eTable 17, a second cell is suppressed as well. The annual percent change is estimated across all years and is reported.*

**eTable 5h. Temporal trend in ECMO by MHD group, 2016–2023.**

| **Group** | **2016** | **2017** | **2018** | **2019** | **2020** | **2021** | **2022** | **2023** | **APC** | **95% CI** | **P** | **P-int** |
| --- | --- | --- | --- | --- | --- | --- | --- | --- | --- | --- | --- | --- |
| Overall | 0.2 | 0.2 | 0.3 | 0.3 | 0.3 | 0.3 | 0.3 | 0.4 | 11.0 | 8.4, 13.7 | <0.001 |  |
| No MHD | 0.2 | 0.2 | 0.3 | 0.3 | 0.3 | 0.3 | 0.4 | 0.4 | 11.3 | 8.6, 14.1 | <0.001 |  |
| Major depression | NR | NR | 0.1 | 0.1 | 0.2 | 0.1 | 0.1 | 0.3 | 14.4 | 5.8, 23.8 | <0.001 | 0.443 |
| Bipolar disorder | NR | NR | NR | NR | NR | NR | NR | NR | NR | NR | NR | NR |
| Anxiety disorders | 0.1 | 0.1 | 0.2 | 0.1 | 0.2 | 0.2 | 0.1 | 0.2 | 7.7 | 0.8, 15.2 | 0.028 | 0.327 |
| Schizophrenia/psychotic | NR | NR | NR | NR | NR | NR | NR | NR | NR | NR | NR | NR |
| PTSD | NR | NR | NR | NR | NR | NR | NR | NR | NR | NR | NR | NR |

*Rates (%) by year; APC and MHD×year interaction (P-int). NR, not reportable: the underlying cell contains 10 or fewer unweighted discharges, so the corresponding percentage is suppressed in accordance with the HCUP Data Use Agreement (see eTable 17). Bipolar disorder is suppressed on the same basis: with 18 ECMO discharges across eight years, every annual cell falls at or below the threshold. The 2016 cell for major depression contains 10 or fewer discharges; the 2017 cell is suppressed additionally so that the 2016 value cannot be recovered from the group total reported in eTable 17.*

**eTable 6a. Age-stratified primary outcomes, 18–49 y.**

| **Outcome** | **Major depression** | **Anxiety disorders** | **Bipolar disorder** | **Schizophrenia/psychotic** | **PTSD** |
| --- | --- | --- | --- | --- | --- |
| Any revascularization | *0.97 (0.91-1.02): P=.22* | *0.99 (0.95-1.03): P=.63* | *1.01 (0.92-1.11): P=.79* | 0.58 (0.50-0.67)*: P<.001* | *0.89 (0.79-1.01): P=.06* |
| PCI | *0.98 (0.93-1.03): P=.37* | 0.91 (0.87-0.94)*: P<.001* | *1.02 (0.93-1.12): P=.66* | 0.61 (0.52-0.70)*: P<.001* | *0.89 (0.78-1.00): P=.05* |
| CABG | *0.99 (0.89-1.09): P=.78* | 1.32 (1.23-1.43)*: P<.001* | *0.95 (0.80-1.14): P=.60* | *0.76 (0.57-1.01): P=.06* | *1.01 (0.81-1.27): P=.91* |
| In-hospital mortality | *0.92 (0.74-1.14): P=.43* | 0.66 (0.54-0.79)*: P<.001* | *0.90 (0.65-1.26): P=.54* | *0.82 (0.49-1.34): P=.42* | *0.90 (0.55-1.47): P=.67* |
| Any MCS | *0.99 (0.87-1.13): P=.89* | *0.97 (0.87-1.09): P=.63* | *1.01 (0.81-1.26): P=.94* | 0.65 (0.44-0.95)*: P=.03* | *0.87 (0.63-1.21): P=.41* |
| IABP | *1.01 (0.87-1.18): P=.89* | *1.02 (0.90-1.15): P=.80* | *1.00 (0.77-1.28): P=.98* | *0.82 (0.55-1.22): P=.32* | *0.84 (0.57-1.22): P=.36* |
| Percutaneous LVAD | *0.96 (0.74-1.24): P=.75* | *0.88 (0.71-1.08): P=.22* | *1.20 (0.81-1.77): P=.36* | *NR* | *0.87 (0.48-1.58): P=.65* |
| ECMO | *0.76 (0.46-1.24): P=.27* | *0.83 (0.56-1.24): P=.36* | *NR* | NE | *NR* |

*Adjusted OR (95% CI): P value. Reference: hospitalizations without the respective disorder. NE, not estimable (too few events). NR, not reportable: the underlying cell contains 10 or fewer unweighted discharges and is therefore suppressed in accordance with the HCUP Data Use Agreement (see eTable 17).*

**eTable 6b. Age-stratified primary outcomes, 50–75 y.**

| **Outcome** | **Major depression** | **Anxiety disorders** | **Bipolar disorder** | **Schizophrenia/psychotic** | **PTSD** |
| --- | --- | --- | --- | --- | --- |
| Any revascularization | 0.92 (0.90-0.94)*: P<.001* | *0.98 (0.97-1.00): P=.12* | 0.78 (0.75-0.82)*: P<.001* | 0.54 (0.51-0.57)*: P<.001* | *0.94 (0.88-1.00): P=.04* |
| PCI | 0.97 (0.95-0.99)*: P=.001* | 0.91 (0.89-0.93)*: P<.001* | 0.83 (0.80-0.88)*: P<.001* | 0.61 (0.57-0.65)*: P<.001* | *0.94 (0.89-1.00): P=.04* |
| CABG | 0.90 (0.87-0.93)*: P<.001* | 1.22 (1.19-1.25)*: P<.001* | 0.83 (0.77-0.90)*: P<.001* | 0.69 (0.61-0.77)*: P<.001* | *0.99 (0.90-1.08): P=.80* |
| In-hospital mortality | 0.81 (0.76-0.85)*: P<.001* | 0.73 (0.69-0.77)*: P<.001* | 0.65 (0.56-0.75)*: P<.001* | *1.11 (0.95-1.29): P=.19* | 0.66 (0.54-0.81)*: P<.001* |
| Any MCS | 0.90 (0.86-0.94)*: P<.001* | *1.03 (0.99-1.07): P=.13* | 0.70 (0.62-0.78)*: P<.001* | 0.85 (0.74-0.97)*: P=.02* | 0.79 (0.69-0.91)*: P=.001* |
| IABP | 0.93 (0.88-0.97)*: P=.002* | *1.05 (1.00-1.09): P=.05* | 0.71 (0.63-0.82)*: P<.001* | *0.87 (0.74-1.02): P=.08* | *0.89 (0.77-1.04): P=.13* |
| Percutaneous LVAD | 0.86 (0.79-0.93)*: P<.001* | *0.97 (0.90-1.05): P=.45* | 0.66 (0.53-0.83)*: P<.001* | *0.84 (0.66-1.08): P=.17* | 0.50 (0.36-0.69)*: P<.001* |
| ECMO | *0.83 (0.67-1.02): P=.07* | 0.82 (0.68-0.99)*: P=.04* | *NR* | NR | *NR* |

*Adjusted OR (95% CI): P value. Reference: hospitalizations without the respective disorder. NE, not estimable (too few events). NR, not reportable: the underlying cell contains 10 or fewer unweighted discharges and is therefore suppressed in accordance with the HCUP Data Use Agreement (see eTable 17).*

**eTable 6c. Age-stratified primary outcomes, ≥76 y.**

| **Outcome** | **Major depression** | **Anxiety disorders** | **Bipolar disorder** | **Schizophrenia/psychotic** | **PTSD** |
| --- | --- | --- | --- | --- | --- |
| Any revascularization | 0.94 (0.91-0.97)*: P<.001* | *1.02 (0.99-1.05): P=.19* | 0.82 (0.71-0.95)*: P=.010* | 0.68 (0.58-0.79)*: P<.001* | *1.05 (0.89-1.25): P=.56* |
| PCI | 0.96 (0.93-0.99)*: P=.02* | *0.98 (0.95-1.01): P=.30* | 0.80 (0.69-0.93)*: P=.005* | 0.63 (0.53-0.74)*: P<.001* | *1.05 (0.88-1.26): P=.55* |
| CABG | 0.91 (0.85-0.97)*: P=.004* | 1.17 (1.10-1.24)*: P<.001* | *1.12 (0.84-1.48): P=.45* | *1.22 (0.91-1.63): P=.19* | *1.02 (0.76-1.39): P=.88* |
| In-hospital mortality | 0.86 (0.81-0.91)*: P<.001* | 0.87 (0.82-0.92)*: P<.001* | *0.92 (0.72-1.17): P=.48* | *0.95 (0.76-1.19): P=.65* | *0.71 (0.49-1.01): P=.06* |
| Any MCS | 0.85 (0.78-0.92)*: P<.001* | *0.98 (0.92-1.06): P=.67* | *0.96 (0.69-1.34): P=.82* | *1.13 (0.83-1.54): P=.43* | *1.00 (0.69-1.45): P=.99* |
| IABP | 0.87 (0.79-0.95)*: P=.003* | *1.03 (0.95-1.12): P=.42* | *1.06 (0.73-1.54): P=.77* | *1.28 (0.91-1.81): P=.16* | *0.95 (0.60-1.49): P=.81* |
| Percutaneous LVAD | 0.79 (0.69-0.90)*: P<.001* | *0.90 (0.80-1.02): P=.11* | *0.76 (0.41-1.40): P=.37* | *NR* | *1.10 (0.61-1.96): P=.76* |
| ECMO | *0.93 (0.48-1.77): P=.81* | *0.78 (0.41-1.50): P=.45* | NE | NE | NE |

*Adjusted OR (95% CI): P value. Reference: hospitalizations without the respective disorder. NE, not estimable (too few events). NR, not reportable: the underlying cell contains 10 or fewer unweighted discharges and is therefore suppressed in accordance with the HCUP Data Use Agreement (see eTable 17).*

**eTable 7. Sensitivity analysis (2016–2022) additionally adjusted for race/ethnicity and hospital region.**

| **Disorder** | **Revasc.** | **PCI** | **CABG** | **Mortality** | **Any MCS** | **IABP** | **pLVAD** | **ECMO** |
| --- | --- | --- | --- | --- | --- | --- | --- | --- |
| Major depression | 0.90 (0.89-0.92)*: P<.001* | 0.94 (0.92-0.95)*: P<.001* | 0.93 (0.90-0.95)*: P<.001* | 0.86 (0.82-0.90)*: P<.001* | 0.90 (0.86-0.93)*: P<.001* | 0.92 (0.88-0.96)*: P<.001* | 0.85 (0.79-0.91)*: P<.001* | 0.77 (0.62-0.95)*: P=.01* |
| Anxiety disorders | 0.94 (0.93-0.96)*: P<.001* | 0.89 (0.87-0.90)*: P<.001* | 1.19 (1.16-1.22)*: P<.001* | 0.81 (0.78-0.85)*: P<.001* | *1.02 (0.99-1.06): P=.20* | *1.05 (1.00-1.09): P=.03* | *0.95 (0.89-1.02): P=.14* | 0.82 (0.68-0.99)*: P=.04* |
| Bipolar disorder | 0.77 (0.74-0.81)*: P<.001* | 0.81 (0.78-0.85)*: P<.001* | 0.88 (0.81-0.95)*: P<.001* | 0.83 (0.73-0.94)*: P=.003* | 0.79 (0.71-0.87)*: P<.001* | 0.79 (0.70-0.89)*: P<.001* | 0.78 (0.64-0.94)*: P=.01* | *0.76 (0.46-1.25): P=.28* |
| Schizophrenia/psychotic | 0.59 (0.56-0.63)*: P<.001* | 0.63 (0.59-0.67)*: P<.001* | 0.81 (0.73-0.90)*: P<.001* | *1.10 (0.96-1.25): P=.17* | *0.95 (0.84-1.08): P=.42* | *1.02 (0.89-1.18): P=.76* | *0.85 (0.67-1.08): P=.19* | NR |
| PTSD | *0.94 (0.89-1.00): P=.04* | 0.94 (0.88-0.99)*: P=.02* | *1.01 (0.93-1.10): P=.80* | 0.65 (0.54-0.78)*: P<.001* | *0.88 (0.77-1.00): P=.06* | *0.95 (0.83-1.10): P=.51* | 0.64 (0.49-0.85)*: P=.002* | *NR* |

*Adjusted OR (95% CI): P value. Same design as the main model, plus race/ethnicity and hospital region (both suppressed in NIS 2023 and therefore excluded from the main 2016–2023 model). NR, not reportable: the underlying cell contains 10 or fewer unweighted discharges and is therefore suppressed in accordance with the HCUP Data Use Agreement (see eTable 17).*

**eTable 8. Expanded in-hospital mortality model additionally adjusted for treatment, complications, and acute organ failure.**

| **Disorder** | **Expanded-model aOR (95% CI)** |
| --- | --- |
| Major depression | 0.89 (0.85-0.94)*: P<.001* |
| Anxiety disorders | 0.80 (0.77-0.84)*: P<.001* |
| Bipolar disorder | *0.88 (0.76-1.02): P=.10* |
| Schizophrenia/psychotic | *0.86 (0.73-1.01): P=.07* |
| PTSD | 0.75 (0.61-0.91)*: P=.004* |

*Adjusted OR (95% CI): P value. This model conditions on intervening (mediating) variables; because adjusting for mediators can introduce overadjustment and collider bias, it is hypothesis-generating rather than causal.*

**eTable 9. Full complication and process-of-care outcome panel — race/ethnicity- and region-adjusted sensitivity model (2016–2022).**

| **Outcome** | **Major depression** | **Anxiety disorders** | **Bipolar disorder** | **Schizophrenia/psychotic** | **PTSD** |
| --- | --- | --- | --- | --- | --- |
| Any revascularization | 0.90 (0.89-0.92)*: P<.001* | 0.94 (0.93-0.96)*: P<.001* | 0.77 (0.74-0.81)*: P<.001* | 0.59 (0.56-0.63)*: P<.001* | *0.94 (0.89-1.00): P=.04* |
| PCI | 0.94 (0.92-0.95)*: P<.001* | 0.89 (0.87-0.90)*: P<.001* | 0.81 (0.78-0.85)*: P<.001* | 0.63 (0.59-0.67)*: P<.001* | 0.94 (0.88-0.99)*: P=.02* |
| CABG | 0.93 (0.90-0.95)*: P<.001* | 1.19 (1.16-1.22)*: P<.001* | 0.88 (0.81-0.95)*: P<.001* | 0.81 (0.73-0.90)*: P<.001* | *1.01 (0.93-1.10): P=.80* |
| Invasive management | 0.94 (0.93-0.96)*: P<.001* | *0.99 (0.97-1.00): P=.14* | 0.81 (0.77-0.85)*: P<.001* | 0.54 (0.51-0.57)*: P<.001* | *1.01 (0.94-1.07): P=.86* |
| Coronary angiography | 0.96 (0.94-0.98)*: P<.001* | *1.00 (0.98-1.02): P=.92* | 0.82 (0.79-0.86)*: P<.001* | 0.57 (0.54-0.61)*: P<.001* | *1.00 (0.94-1.06): P=1.00* |
| Left heart catheterization | 0.94 (0.92-0.96)*: P<.001* | *1.00 (0.98-1.01): P=.83* | 0.84 (0.81-0.88)*: P<.001* | 0.63 (0.59-0.66)*: P<.001* | *0.94 (0.89-1.00): P=.05* |
| Right heart catheterization | *0.96 (0.92-1.00): P=.04* | *0.96 (0.92-1.00): P=.05* | *0.96 (0.86-1.07): P=.44* | 0.80 (0.69-0.93)*: P=.002* | *0.93 (0.81-1.08): P=.34* |
| Coronary thrombectomy | 0.95 (0.92-0.99)*: P=.02* | 0.96 (0.93-0.99)*: P=.03* | 0.90 (0.81-0.99)*: P=.02* | 0.74 (0.65-0.85)*: P<.001* | *0.88 (0.78-1.00): P=.05* |
| Intravascular ultrasound | *1.02 (0.98-1.06): P=.31* | *0.99 (0.95-1.02): P=.40* | 0.87 (0.79-0.96)*: P=.004* | 0.82 (0.72-0.93)*: P=.002* | 1.28 (1.16-1.41)*: P<.001* |
| Fibrinolysis | *0.91 (0.81-1.01): P=.07* | *1.10 (1.00-1.21): P=.05* | *0.94 (0.72-1.22): P=.64* | *0.92 (0.66-1.30): P=.65* | *1.03 (0.74-1.43): P=.86* |
| In-hospital mortality | 0.86 (0.82-0.90)*: P<.001* | 0.81 (0.78-0.85)*: P<.001* | 0.83 (0.73-0.94)*: P=.003* | *1.10 (0.96-1.25): P=.17* | 0.65 (0.54-0.78)*: P<.001* |
| Any MCS | 0.90 (0.86-0.93)*: P<.001* | *1.02 (0.99-1.06): P=.20* | 0.79 (0.71-0.87)*: P<.001* | *0.95 (0.84-1.08): P=.42* | *0.88 (0.77-1.00): P=.06* |
| IABP | 0.92 (0.88-0.96)*: P<.001* | *1.05 (1.00-1.09): P=.03* | 0.79 (0.70-0.89)*: P<.001* | *1.02 (0.89-1.18): P=.76* | *0.95 (0.83-1.10): P=.51* |
| Percutaneous LVAD | 0.85 (0.79-0.91)*: P<.001* | *0.95 (0.89-1.02): P=.14* | 0.78 (0.64-0.94)*: P=.01* | *0.85 (0.67-1.08): P=.19* | 0.64 (0.49-0.85)*: P=.002* |
| ECMO | 0.77 (0.62-0.95)*: P=.01* | 0.82 (0.68-0.99)*: P=.03* | *0.76 (0.46-1.25): P=.28* | NR | *NR* |
| Durable LVAD | 1.83 (1.20-2.79)*: P=.005* | 0.59 (0.36-0.98)*: P=.04* | NE | *1.27 (0.35-4.61): P=.72* | NE |
| Heart transplant | NE | *1.44 (0.46-4.50): P=.53* | NE | NE | NE |
| Invasive mechanical ventilation | 0.87 (0.84-0.91)*: P<.001* | 0.91 (0.87-0.94)*: P<.001* | 0.85 (0.77-0.93)*: P<.001* | 1.39 (1.26-1.54)*: P<.001* | 0.86 (0.75-0.98)*: P=.02* |
| Noninvasive ventilation | 1.07 (1.03-1.12)*: P=.002* | 1.23 (1.18-1.28)*: P<.001* | *1.06 (0.94-1.19): P=.32* | 1.21 (1.06-1.39)*: P=.004* | *0.96 (0.82-1.12): P=.61* |
| Acute hemodialysis | *0.99 (0.94-1.05): P=.82* | *0.97 (0.92-1.03): P=.33* | 0.80 (0.69-0.93)*: P=.005* | *0.93 (0.78-1.11): P=.42* | 0.70 (0.55-0.88)*: P=.002* |
| Blood transfusion | *1.01 (0.97-1.05): P=.67* | 1.05 (1.01-1.10)*: P=.009* | *0.89 (0.79-1.00): P=.06* | 1.16 (1.02-1.32)*: P=.03* | 0.83 (0.71-0.98)*: P=.03* |
| Any complication | *0.98 (0.96-1.00): P=.04* | 0.95 (0.94-0.97)*: P<.001* | *0.98 (0.93-1.04): P=.58* | 1.25 (1.17-1.34)*: P<.001* | *0.95 (0.89-1.02): P=.18* |
| Cardiogenic shock | 0.91 (0.88-0.94)*: P<.001* | *0.98 (0.95-1.01): P=.21* | 0.83 (0.76-0.92)*: P<.001* | *1.05 (0.94-1.17): P=.43* | 0.83 (0.73-0.95)*: P=.006* |
| Cardiac arrest | 0.83 (0.79-0.87)*: P<.001* | 0.88 (0.84-0.92)*: P<.001* | 0.79 (0.70-0.90)*: P<.001* | 1.18 (1.02-1.35)*: P=.02* | *1.00 (0.86-1.17): P=.97* |
| Acute kidney injury | *0.99 (0.97-1.01): P=.29* | 0.97 (0.95-0.99)*: P=.002* | *1.02 (0.96-1.08): P=.60* | 1.27 (1.18-1.36)*: P<.001* | *0.95 (0.89-1.03): P=.23* |
| Major bleeding | *0.98 (0.90-1.06): P=.58* | 0.87 (0.80-0.94)*: P=.001* | *1.05 (0.84-1.31): P=.66* | *1.26 (0.99-1.60): P=.06* | 0.61 (0.41-0.89)*: P=.010* |
| Ischemic stroke | 1.13 (1.05-1.21)*: P<.001* | 0.79 (0.73-0.84)*: P<.001* | *0.90 (0.74-1.10): P=.29* | *1.15 (0.94-1.42): P=.18* | 0.67 (0.50-0.90)*: P=.009* |
| Intracranial hemorrhage | *1.08 (0.89-1.31): P=.46* | 0.74 (0.61-0.91)*: P=.005* | *0.99 (0.59-1.66): P=.98* | *0.98 (0.55-1.73): P=.94* | *0.80 (0.37-1.71): P=.56* |
| Acute respiratory failure | *0.98 (0.96-1.00): P=.11* | 1.08 (1.06-1.10)*: P<.001* | 0.92 (0.86-0.97)*: P=.006* | 1.22 (1.13-1.31)*: P<.001* | 0.85 (0.78-0.93)*: P<.001* |
| Acute hepatic failure | 0.69 (0.61-0.76)*: P<.001* | 0.73 (0.66-0.81)*: P<.001* | 0.52 (0.41-0.66)*: P<.001* | 0.71 (0.53-0.94)*: P=.02* | 0.58 (0.41-0.83)*: P=.003* |
| Hematologic failure/DIC | *0.98 (0.95-1.02): P=.42* | 1.07 (1.04-1.11)*: P<.001* | *0.96 (0.86-1.07): P=.48* | *1.12 (0.99-1.27): P=.07* | *0.90 (0.78-1.03): P=.12* |
| Acute neurologic failure | 1.13 (1.08-1.18)*: P<.001* | *1.00 (0.96-1.04): P=.87* | 1.21 (1.10-1.34)*: P<.001* | 2.10 (1.91-2.30)*: P<.001* | *0.92 (0.79-1.06): P=.24* |
| Palliative-care encounter | *1.03 (0.98-1.08): P=.27* | 1.12 (1.07-1.17)*: P<.001* | *1.01 (0.88-1.17): P=.86* | 1.27 (1.10-1.46)*: P<.001* | 0.79 (0.64-0.97)*: P=.02* |
| DNR status | 1.25 (1.21-1.29)*: P<.001* | 1.05 (1.02-1.09)*: P<.001* | 1.29 (1.17-1.42)*: P<.001* | 1.39 (1.25-1.54)*: P<.001* | *0.99 (0.87-1.12): P=.86* |
| Access-site complication | *1.04 (0.89-1.21): P=.64* | *0.95 (0.82-1.10): P=.51* | *1.16 (0.79-1.71): P=.46* | *1.51 (0.96-2.37): P=.08* | *1.02 (0.59-1.76): P=.94* |
| Acute limb ischemia | *0.89 (0.78-1.01): P=.08* | *1.00 (0.88-1.12): P=.95* | *0.78 (0.55-1.11): P=.17* | *0.94 (0.61-1.45): P=.79* | *0.85 (0.53-1.36): P=.51* |
| Hemolysis | *0.84 (0.40-1.75): P=.64* | *1.53 (0.87-2.68): P=.14* | NE | NE | NE |

*Adjusted OR (95% CI): P value for every outcome; reference = hospitalizations without the respective disorder. NR, not reportable: the underlying cell contains 10 or fewer unweighted discharges and is therefore suppressed in accordance with the HCUP Data Use Agreement (see eTable 17).*

**eTable 10a. Primary outcomes with a COVID-era covariate (2016–2023).**

| **Outcome** | **Major depression** | **Anxiety disorders** | **Bipolar disorder** | **Schizophrenia/psychotic** | **PTSD** |
| --- | --- | --- | --- | --- | --- |
| Any revascularization | 0.94 (0.92-0.95)*: P<.001* | *0.98 (0.97-1.00): P=.04* | 0.81 (0.78-0.85)*: P<.001* | 0.56 (0.53-0.60)*: P<.001* | 0.93 (0.88-0.98)*: P=.008* |
| PCI | 0.97 (0.95-0.98)*: P<.001* | 0.92 (0.90-0.93)*: P<.001* | 0.85 (0.82-0.89)*: P<.001* | 0.61 (0.58-0.65)*: P<.001* | 0.93 (0.88-0.98)*: P=.004* |
| CABG | 0.93 (0.91-0.96)*: P<.001* | 1.22 (1.19-1.25)*: P<.001* | 0.88 (0.82-0.95)*: P<.001* | 0.76 (0.69-0.84)*: P<.001* | *1.01 (0.93-1.09): P=.87* |
| In-hospital mortality | 0.86 (0.82-0.90)*: P<.001* | 0.81 (0.77-0.84)*: P<.001* | 0.81 (0.72-0.91)*: P<.001* | *1.11 (0.98-1.26): P=.09* | 0.68 (0.58-0.81)*: P<.001* |
| Any MCS | 0.91 (0.88-0.94)*: P<.001* | *1.02 (0.98-1.05): P=.31* | 0.78 (0.71-0.86)*: P<.001* | *0.89 (0.79-1.00): P=.05* | 0.82 (0.73-0.93)*: P=.002* |
| IABP | 0.94 (0.90-0.98)*: P=.002* | 1.05 (1.01-1.09)*: P=.01* | 0.81 (0.72-0.90)*: P<.001* | *0.94 (0.83-1.08): P=.41* | *0.90 (0.79-1.03): P=.13* |
| Percutaneous LVAD | 0.85 (0.80-0.91)*: P<.001* | *0.94 (0.88-1.00): P=.04* | 0.76 (0.63-0.90)*: P=.002* | *0.81 (0.65-1.01): P=.06* | 0.59 (0.46-0.77)*: P<.001* |
| ECMO | *0.84 (0.70-1.01): P=.06* | 0.80 (0.67-0.94)*: P=.008* | *0.66 (0.40-1.07): P=.09* | NR | *NR* |

*Adjusted OR (95% CI): P value. NR, not reportable: the underlying cell contains 10 or fewer unweighted discharges and is therefore suppressed in accordance with the HCUP Data Use Agreement (see eTable 17).*

**eTable 10b. Primary outcomes restricted to the pre-COVID period (2016–2019).**

| **Outcome** | **Major depression** | **Anxiety disorders** | **Bipolar disorder** | **Schizophrenia/psychotic** | **PTSD** |
| --- | --- | --- | --- | --- | --- |
| Any revascularization | 0.92 (0.90-0.94)*: P<.001* | *0.99 (0.96-1.01): P=.22* | 0.81 (0.77-0.86)*: P<.001* | 0.57 (0.52-0.61)*: P<.001* | *1.01 (0.93-1.09): P=.86* |
| PCI | 0.95 (0.93-0.98)*: P<.001* | 0.92 (0.90-0.94)*: P<.001* | 0.85 (0.80-0.90)*: P<.001* | 0.60 (0.56-0.65)*: P<.001* | *0.97 (0.90-1.05): P=.44* |
| CABG | 0.93 (0.89-0.96)*: P<.001* | 1.20 (1.16-1.24)*: P<.001* | *0.91 (0.82-1.00): P=.06* | 0.80 (0.70-0.92)*: P=.001* | *1.09 (0.97-1.23): P=.14* |
| In-hospital mortality | 0.89 (0.84-0.94)*: P<.001* | 0.80 (0.75-0.84)*: P<.001* | *0.87 (0.73-1.02): P=.09* | *1.16 (0.97-1.37): P=.09* | 0.59 (0.45-0.77)*: P<.001* |
| Any MCS | 0.90 (0.85-0.95)*: P<.001* | *1.02 (0.97-1.07): P=.38* | 0.80 (0.69-0.92)*: P=.002* | *0.90 (0.76-1.06): P=.20* | *0.83 (0.70-1.00): P=.05* |
| IABP | 0.92 (0.86-0.97)*: P=.003* | *1.05 (1.00-1.11): P=.05* | 0.81 (0.69-0.95)*: P=.01* | *1.02 (0.85-1.23): P=.81* | *0.92 (0.76-1.12): P=.40* |
| Percutaneous LVAD | 0.88 (0.80-0.97)*: P=.01* | *0.91 (0.82-1.00): P=.04* | *0.77 (0.58-1.01): P=.06* | 0.66 (0.45-0.94)*: P=.02* | 0.54 (0.34-0.84)*: P=.007* |
| ECMO | 0.71 (0.52-0.95)*: P=.02* | *0.84 (0.65-1.10): P=.21* | *NR* | *NR* | *NR* |

*Adjusted OR (95% CI): P value. NR, not reportable: the underlying cell contains 10 or fewer unweighted discharges and is therefore suppressed in accordance with the HCUP Data Use Agreement (see eTable 17).*

**eTable 11. Sex-stratified MHD prevalence trends, 2016–2023.**

| **Variable** | **2016** | **2017** | **2018** | **2019** | **2020** | **2021** | **2022** | **2023** | **APC** | **95% CI** | **P** | **P-int** |
| --- | --- | --- | --- | --- | --- | --- | --- | --- | --- | --- | --- | --- |
| Major depression in males | 6.2 | 6.6 | 6.7 | 6.8 | 7.0 | 7.1 | 6.9 | 7.3 | 2.0 | 1.4, 2.5 | <0.001 |  |
| Major depression in females | 12.2 | 12.7 | 12.9 | 13.5 | 14.0 | 14.4 | 14.4 | 15.1 | 3.5 | 2.9, 4.0 | <0.001 | <0.001 |
| Anxiety disorders in males | 7.1 | 7.7 | 7.9 | 8.1 | 8.6 | 9.1 | 8.9 | 9.2 | 3.9 | 3.3, 4.4 | <0.001 |  |
| Anxiety disorders in females | 14.8 | 15.6 | 15.9 | 16.6 | 17.7 | 18.5 | 18.3 | 18.8 | 4.3 | 3.8, 4.9 | <0.001 | 0.109 |
| Bipolar disorder in males | 1.0 | 1.0 | 1.0 | 1.0 | 1.1 | 1.0 | 1.0 | 1.1 | 1.8 | 0.7, 3.0 | 0.002 |  |
| Bipolar disorder in females | 1.3 | 1.4 | 1.6 | 1.6 | 1.8 | 1.8 | 1.9 | 2.0 | 5.8 | 4.6, 7.0 | <0.001 | <0.001 |
| Schizophrenia/psychotic in males | 0.7 | 0.8 | 0.7 | 0.7 | 0.7 | 0.8 | 0.7 | 0.7 | 0.2 | -1.1, 1.6 | 0.745 |  |
| Schizophrenia/psychotic in females | 0.7 | 0.7 | 0.8 | 0.7 | 0.8 | 0.7 | 0.9 | 0.7 | 1.1 | -0.5, 2.8 | 0.171 | 0.378 |
| PTSD in males | 0.6 | 0.7 | 0.7 | 0.9 | 0.9 | 0.9 | 0.9 | 1.1 | 7.2 | 5.8, 8.6 | <0.001 |  |
| PTSD in females | 0.3 | 0.4 | 0.5 | 0.5 | 0.7 | 0.8 | 0.9 | 1.1 | 17.3 | 15.0, 19.7 | <0.001 | <0.001 |

*P-int = MHD×sex interaction P value.*

**eTable 12. Race/ethnicity-stratified MHD prevalence trends, 2016–2022.**

| **Variable** | **2016** | **2017** | **2018** | **2019** | **2020** | **2021** | **2022** | **APC** | **95% CI** | **P** | **P-int** |
| --- | --- | --- | --- | --- | --- | --- | --- | --- | --- | --- | --- |
| Major depression in White | 9.2 | 9.8 | 9.9 | 10.3 | 10.5 | 10.9 | 10.9 | 3.1 | 2.5, 3.7 | <0.001 | <0.001 |
| Major depression in Black | 6.1 | 6.6 | 6.6 | 6.5 | 6.6 | 6.9 | 6.9 | 1.7 | 0.2, 3.2 | 0.025 |  |
| Major depression in Hispanic | 6.9 | 7.2 | 7.3 | 7.3 | 7.4 | 7.6 | 7.0 | 0.5 | -1.2, 2.1 | 0.578 |  |
| Major depression in Asian/PI | 4.6 | 3.9 | 4.0 | 3.6 | 4.3 | 4.0 | 3.5 | -2.8 | -5.9, 0.4 | 0.090 |  |
| Major depression in Native American | 9.9 | 8.8 | 9.2 | 7.5 | 9.0 | 9.8 | 10.6 | 1.8 | -3.8, 7.8 | 0.536 |  |
| Major depression in Other | 5.9 | 6.1 | 5.4 | 6.5 | 7.1 | 6.5 | 6.2 | 2.0 | -0.8, 4.9 | 0.165 |  |
| Anxiety disorders in White | 11.3 | 12.1 | 12.2 | 12.6 | 13.3 | 14.0 | 14.0 | 4.4 | 3.8, 4.9 | <0.001 | 0.245 |
| Anxiety disorders in Black | 6.2 | 6.5 | 7.2 | 7.2 | 8.0 | 8.1 | 7.7 | 4.8 | 3.3, 6.2 | <0.001 |  |
| Anxiety disorders in Hispanic | 8.0 | 8.1 | 8.7 | 9.0 | 8.9 | 10.2 | 9.8 | 4.3 | 2.8, 5.9 | <0.001 |  |
| Anxiety disorders in Asian/PI | 4.1 | 4.3 | 4.4 | 4.1 | 4.7 | 5.4 | 4.7 | 3.6 | 0.3, 6.9 | 0.030 |  |
| Anxiety disorders in Native American | 7.6 | 7.7 | 8.2 | 7.2 | 9.8 | 11.4 | 11.2 | 9.0 | 3.3, 15.1 | 0.002 |  |
| Anxiety disorders in Other | 7.1 | 8.9 | 7.8 | 8.4 | 8.9 | 9.5 | 8.3 | 2.7 | 0.2, 5.3 | 0.038 |  |
| Bipolar disorder in White | 1.2 | 1.3 | 1.3 | 1.3 | 1.5 | 1.4 | 1.5 | 3.5 | 2.3, 4.7 | <0.001 | 0.255 |
| Bipolar disorder in Black | 1.2 | 1.3 | 1.3 | 1.3 | 1.5 | 1.5 | 1.5 | 4.9 | 1.9, 8.0 | 0.001 |  |
| Bipolar disorder in Hispanic | 0.6 | 0.6 | 0.9 | 0.9 | 0.9 | 0.9 | 0.7 | 3.1 | -0.8, 7.2 | 0.126 |  |
| Bipolar disorder in Asian/PI | 0.4 | 0.3 | 0.4 | 0.3 | 0.3 | 0.4 | 0.4 | 1.3 | -9.4, 13.1 | 0.824 |  |
| Bipolar disorder in Native American | 0.3 | 1.1 | 1.1 | 1.2 | 1.9 | 2.0 | 1.6 | 20.1 | 7.1, 34.6 | 0.002 |  |
| Bipolar disorder in Other | 0.9 | 0.8 | 1.0 | 0.9 | 1.1 | 0.7 | 1.0 | 1.0 | -5.4, 8.0 | 0.760 |  |
| Schizophrenia/psychotic in White | 0.6 | 0.6 | 0.6 | 0.6 | 0.6 | 0.6 | 0.6 | 1.4 | -0.3, 3.1 | 0.111 | 0.347 |
| Schizophrenia/psychotic in Black | 1.5 | 1.7 | 1.9 | 1.6 | 1.7 | 1.7 | 1.7 | 1.0 | -1.7, 3.7 | 0.493 |  |
| Schizophrenia/psychotic in Hispanic | 0.8 | 0.7 | 0.8 | 0.7 | 0.9 | 0.9 | 0.8 | 2.2 | -2.1, 6.8 | 0.320 |  |
| Schizophrenia/psychotic in Asian/PI | 0.6 | 0.4 | 0.4 | 0.4 | 0.4 | 0.4 | 0.4 | -4.8 | -13.8, 5.2 | 0.338 |  |
| Schizophrenia/psychotic disorder in Native Americans | 0.1 | 0.6 | 0.8 | 0.8 | 0.5 | 0.6 | 1.0 | 14.0 | -4.4, 36.1 | 0.145 |  |
| Schizophrenia/psychotic in Other | 0.7 | 1.0 | 0.7 | 0.6 | 0.8 | 0.5 | 0.6 | -5.7 | -12.8, 1.8 | 0.134 |  |
| PTSD in White | 0.6 | 0.6 | 0.7 | 0.8 | 0.9 | 0.9 | 1.0 | 9.9 | 8.2, 11.7 | <0.001 | 0.194 |
| PTSD in Black | 0.5 | 0.6 | 0.7 | 0.7 | 0.7 | 0.9 | 1.0 | 10.8 | 6.6, 15.1 | <0.001 |  |
| PTSD in Hispanic | 0.3 | 0.4 | 0.5 | 0.4 | 0.4 | 0.6 | 0.6 | 10.7 | 4.6, 17.2 | <0.001 |  |
| PTSD in Asian/PI | 0.4 | 0.3 | 0.2 | 0.3 | 0.3 | 0.2 | 0.3 | -3.1 | -14.5, 9.9 | 0.627 |  |
| PTSD in Native American | 0.1 | 0.8 | 0.8 | 1.5 | 0.9 | 2.0 | 1.5 | 24.3 | 9.4, 41.2 | <0.001 |  |
| PTSD in Other | 0.2 | 0.4 | 0.5 | 0.4 | 0.6 | 0.8 | 0.5 | 13.2 | 3.9, 23.4 | 0.005 |  |

*Race/ethnicity available only through 2022. P-int = MHD×race interaction P value.*

**eTable 13. MHD×sex and MHD×race interaction P values for the revascularization association.**

| **Disorder** | **P (MHD×sex)** | **P (MHD×race)** |
| --- | --- | --- |
| Major depression | <0.001 | <0.001 |
| Anxiety disorders | 0.109 | 0.245 |
| Bipolar disorder | <0.001 | 0.255 |
| Schizophrenia/psychotic | 0.378 | 0.347 |
| PTSD | <0.001 | 0.194 |

*P values for effect-modification tests on the revascularization association.*

**eTable 14a. Primary outcomes restricted to the incident-event cohort (no prior MI, PCI, or CABG).**

| **Disorder** | **Revasc.** | **PCI** | **Invasive mgmt** | **Angiography** | **Mortality** |
| --- | --- | --- | --- | --- | --- |
| Major depression | 0.93 (0.91–0.94): P<.001 | 0.96 (0.94–0.98): P<.001 | 0.98 (0.96–1.01): P=.14 | 1.00 (0.98–1.02): P=.98 | 0.88 (0.84–0.93): P<.001 |
| Anxiety disorders | 0.97 (0.95–0.99): P=.004 | 0.90 (0.89–0.92): P<.001 | 1.03 (1.01–1.05): P=.005 | 1.04 (1.02–1.06): P<.001 | 0.79 (0.75–0.83): P<.001 |
| Bipolar disorder | 0.84 (0.80–0.89): P<.001 | 0.88 (0.84–0.92): P<.001 | 0.86 (0.82–0.91): P<.001 | 0.88 (0.84–0.93): P<.001 | 0.83 (0.72–0.95): P=.006 |
| Schizophrenia/psychotic | 0.54 (0.50–0.57): P<.001 | 0.59 (0.55–0.63): P<.001 | 0.50 (0.47–0.53): P<.001 | 0.54 (0.51–0.57): P<.001 | 1.12 (0.97–1.28): P=.13 |
| PTSD | 0.96 (0.90–1.03): P=.23 | 0.97 (0.91–1.03): P=.30 | 1.04 (0.96–1.12): P=.32 | 1.03 (0.96–1.11): P=.36 | 0.69 (0.57–0.84): P<.001 |

*Adjusted OR (95% CI): P value. n = 669,715 unweighted hospitalizations. Same specification as the main model, with prior MI, prior PCI, and prior CABG omitted (constant within the domain). Fitted on the full survey design by domain estimation, not by subsetting before weighting.*

**eTable 14b. Primary outcomes in hospitalizations with established coronary disease (any prior MI, PCI, or CABG).**

| **Disorder** | **Revasc.** | **PCI** | **Invasive mgmt** | **Angiography** | **Mortality** |
| --- | --- | --- | --- | --- | --- |
| Major depression | 0.96 (0.93–0.98): P=.002 | 0.99 (0.96–1.02): P=.37 | 0.97 (0.94–1.00): P=.05 | 0.98 (0.95–1.01): P=.21 | 0.80 (0.74–0.86): P<.001 |
| Anxiety disorders | 1.02 (0.99–1.05): P=.18 | 0.95 (0.93–0.98): P<.001 | 1.00 (0.97–1.02): P=.75 | 1.00 (0.98–1.03): P=.75 | 0.86 (0.80–0.92): P<.001 |
| Bipolar disorder | 0.76 (0.71–0.82): P<.001 | 0.80 (0.75–0.86): P<.001 | 0.82 (0.76–0.89): P<.001 | 0.83 (0.77–0.90): P<.001 | 0.76 (0.60–0.98): P=.03 |
| Schizophrenia/psychotic | 0.67 (0.60–0.74): P<.001 | 0.69 (0.62–0.77): P<.001 | 0.58 (0.52–0.64): P<.001 | 0.61 (0.55–0.67): P<.001 | 1.10 (0.85–1.42): P=.49 |
| PTSD | 0.91 (0.83–0.99): P=.04 | 0.87 (0.80–0.96): P=.003 | 0.95 (0.87–1.05): P=.35 | 0.96 (0.88–1.06): P=.44 | 0.67 (0.49–0.91): P=.01 |

*Adjusted OR (95% CI): P value. n = 282,793 unweighted hospitalizations. Specification as in eTable 14a.*

**eTable 15. Mental health disorder × prior-coronary-disease interaction, full cohort.**

| **Disorder** | **Revasc.** | **PCI** | **Invasive mgmt** | **Angiography** | **Mortality** |
| --- | --- | --- | --- | --- | --- |
| Major depression | 1.08 (1.04–1.12): P<.001 | 1.07 (1.04–1.11): P<.001 | 1.00 (0.96–1.04): P=.95 | 1.00 (0.96–1.03): P=.80 | 0.90 (0.82–0.99): P=.02 |
| Anxiety disorders | 1.12 (1.09–1.16): P<.001 | 1.09 (1.06–1.13): P<.001 | 0.97 (0.94–1.00): P=.09 | 0.97 (0.94–1.00): P=.08 | 1.10 (1.01–1.19): P=.04 |
| Bipolar disorder | 0.98 (0.90–1.08): P=.72 | 0.94 (0.86–1.03): P=.18 | 0.96 (0.87–1.05): P=.36 | 0.95 (0.87–1.04): P=.27 | 0.93 (0.70–1.23): P=.62 |
| Schizophrenia/psychotic | 1.29 (1.14–1.46): P<.001 | 1.18 (1.04–1.34): P=.01 | 1.17 (1.04–1.32): P=.01 | 1.15 (1.02–1.29): P=.02 | 0.97 (0.72–1.30): P=.82 |
| PTSD | 0.94 (0.84–1.05): P=.28 | 0.90 (0.80–1.01): P=.06 | 0.94 (0.83–1.06): P=.32 | 0.96 (0.85–1.08): P=.47 | 0.94 (0.65–1.35): P=.73 |
| **Joint Wald test (5 df)** | P<.001 | P<.001 | P=.05 | P=.07 | P=.16 |

*Ratio of adjusted odds ratios (95% CI): P value, for each disorder × prior coronary disease interaction term; values above 1.0 indicate that the disorder-associated deficit is smaller among patients with established coronary disease. The three prior-revascularization indicators are replaced by a single prior-coronary-disease term to avoid collinearity with the interaction. The final row gives the joint Wald test across all five interaction terms (5 df). n = 952,508 unweighted hospitalizations.*

**eTable 16. Parsimonious covariate model.**

| **Disorder** | **Revasc.** | **PCI** | **Invasive mgmt** | **Angiography** | **Mortality** |
| --- | --- | --- | --- | --- | --- |
| Major depression | 0.96 (0.95–0.98): P<.001 | 0.98 (0.96–0.99): P=.003 | 1.00 (0.98–1.02): P=.87 | 1.01 (1.00–1.03): P=.12 | 0.82 (0.78–0.85): P<.001 |
| Anxiety disorders | 1.00 (0.98–1.01): P=.64 | 0.93 (0.91–0.94): P<.001 | 1.04 (1.02–1.05): P<.001 | 1.05 (1.03–1.06): P<.001 | 0.77 (0.74–0.80): P<.001 |
| Bipolar disorder | 0.81 (0.78–0.85): P<.001 | 0.84 (0.81–0.88): P<.001 | 0.84 (0.80–0.88): P<.001 | 0.86 (0.82–0.90): P<.001 | 0.82 (0.73–0.92): P<.001 |
| Schizophrenia/psychotic | 0.54 (0.51–0.57): P<.001 | 0.59 (0.56–0.62): P<.001 | 0.50 (0.47–0.52): P<.001 | 0.53 (0.50–0.56): P<.001 | 1.20 (1.06–1.36): P=.003 |
| PTSD | 0.94 (0.89–0.99): P=.01 | 0.93 (0.88–0.98): P=.006 | 1.01 (0.95–1.07): P=.81 | 1.01 (0.95–1.07): P=.72 | 0.66 (0.56–0.78): P<.001 |

*Adjusted OR (95% CI): P value. Covariates restricted to age, sex, primary payer, income quartile, rural residence, hospital bed size, hospital location/teaching status, MI type, anemia, chronic liver disease, chronic kidney disease, dialysis dependence, dementia, peripheral arterial disease, atrial fibrillation/flutter, alcohol use disorder, drug use disorder, and prior CABG — that is, to variables plausibly influencing both psychiatric diagnosis coding and the decision to pursue an invasive strategy. Main-model estimates are given in Table 2 for comparison. n = 952,508 unweighted hospitalizations.*

**eTable 16 footnote — comparison with the main model (schizophrenia/psychotic disorders).**

| **Model** | **Revasc.** | **PCI** | **Invasive mgmt** | **Angiography** | **Mortality** |
| --- | --- | --- | --- | --- | --- |
| Main model | 0.56 (0.53–0.60): P<.001 | 0.61 (0.58–0.65): P<.001 | 0.52 (0.49–0.55): P<.001 | 0.55 (0.53–0.58): P<.001 | 1.11 (0.98–1.26): P=.09 |
| Parsimonious | 0.54 (0.51–0.57): P<.001 | 0.59 (0.56–0.62): P<.001 | 0.50 (0.47–0.52): P<.001 | 0.53 (0.50–0.56): P<.001 | 1.20 (1.06–1.36): P=.003 |

*Procedural estimates are stable across specifications; the in-hospital mortality estimate is not, and no inference about in-hospital mortality is drawn from it.*

**eTable 17. Unweighted mechanical circulatory support event counts by mental health disorder group.**

| **Group** | **Hospitalizations (unweighted)** | **IABP** | **pLVAD** | **ECMO** | **Any MCS** |
| --- | --- | --- | --- | --- | --- |
| No MHD | 792,628 | 32,823 | 13,959 | 2,263 | 45,819 |
| Major depression | 90,311 | 2,977 | 1,091 | 135 | 3,991 |
| Anxiety disorders | 111,446 | 3,882 | 1,374 | 168 | 5,162 |
| Bipolar disorder | 12,280 | 353 | 131 | 18 | 478 |
| Schizophrenia/psychotic | 7,143 | 243 | 89 | ≤10* | 323 |
| PTSD | 7,316 | 244 | 65 | ≤10* | 309 |

** Cells containing 10 or fewer discharges are suppressed in accordance with the HCUP Data Use Agreement. Counts are unweighted and groups overlap, since disorders may co-occur within the same hospitalization. Device-level estimates for extracorporeal membrane oxygenation in the schizophrenia/psychotic and PTSD groups are not reported for this reason.*
